# N(6)-methyladenosine methylation-regulated polo-like kinase 1 cell cycle homeostasis as a potential target of radiotherapy in pancreatic adenocarcinoma

**DOI:** 10.1038/s41598-022-15196-5

**Published:** 2022-06-30

**Authors:** Shotaro Tatekawa, Keisuke Tamari, Ryota Chijimatsu, Masamitsu Konno, Daisuke Motooka, Suguru Mitsufuji, Hirofumi Akita, Shogo Kobayashi, Yoshiki Murakumo, Yuichiro Doki, Hidetoshi Eguchi, Hideshi Ishii, Kazuhiko Ogawa

**Affiliations:** 1 Department of Radiation Oncology, Osaka University Graduate School of Medicine, Yamadaoka 2-2, Suita, Osaka 565-0871 Japan; 2 Department of Medical Data Science, Center of Medical Innovation and Translational Research, Osaka University Graduate School of Medicine, Yamadaoka 2-2, Suita, Osaka 565-0871 Japan; 3 Genome Information Research Center, Research Institute for Microbial Diseases, Osaka University, Suita, Osaka 565-0871 Japan; 4 Department of Gastroenterological Surgery, Osaka University Graduate School of Medicine, Suita, Osaka 565-0871 Japan; 5 Department of Pathology, Kitasato University School of Medicine, Sagamihara, Kanagawa 252-0374 Japan; 6 Present Address: Division of Tumor Biology, Research Institute for Biomedical Sciences (RIBS), Tokyo University of Science, Noda, Chiba Japan

**Keywords:** RNA modification, Pancreatic cancer

## Abstract

In pancreatic cancer, methyltransferase-like 3 (METTL3), a N(6)-methyladenosine (m6A) methyltransferase, has a favorable effect on tumors and is a risk factor for patients’ prognosis. However, the details of what genes are regulated by METTL3 remain unknown. Several RNAs are methylated, and what genes are favored in pancreatic cancer remains unclear. By epitranscriptomic analysis, we report that polo-like kinase 1 (PLK1) is an important hub gene defining patient prognosis in pancreatic cancer and that RNA methylation is involved in regulating its cell cycle-specific expression. We found that insulin like growth factor 2 mRNA binding protein 2 (IGF2BP2) binds to m6A of PLK1 3′ untranslated region and is involved in upregulating PLK1 expression and that demethylation of this site activates the ataxia telangiectasia and Rad3-related protein pathway by replicating stress and increasing mitotic catastrophe, resulting in increased radiosensitivity. This suggests that PLK1 methylation is essential for cell cycle maintenance in pancreatic cancer and is a new therapeutic target.

## Introduction

As pancreatic cancer is often advanced stage at the time of diagnosis due to late onset of symptoms, most patients are diagnosed with unresectable locally advanced or metastatic disease. In addition, pancreatic adenocarcinoma is highly resistant to all existing therapies, including radiation therapy, and it is one of the cancers with the poorest prognosis^1,2^. Pancreatic adenocarcinomas have some frequent genetic abnormalities in the mutational activation of the Kirsten rat sarcoma viral oncogene homolog (KRAS) oncogene; inactivation of tumor suppressor genes, including cyclin dependent kinase inhibitor 2A (CDKN2A), tumor protein P53 (TP53), mothers against decapentaplegic homolog 4 (SMAD4), and breast cancer type 2 susceptibility protein (BRCA2). In addition to these, there are recognized widespread chromosomal losses, gene amplifications, and telomere shortening^3,4^. Despite the recent molecular pathological elucidation in pancreatic adenocarcinoma, it has not directly led to the development of effective treatments. Therefore, new therapeutic strategies are needed to eradicate pancreatic adenocarcinoma, and the search for new therapeutic targets is an urgent issue.

More than 100 chemical modifications of RNA have been discovered, among which N6-methyladenosine (m6A) is the most common modification on the epitranscriptome of mRNA and is introduced by the human methyltransferase complex consisting of methyltransferase-like 3 (METTL3) and METTL14 and the Wilms tumor 1-associated protein complex. m6A modification plays an important role in the regulation of mRNA stability, decay, nuclear retention, and translation through various m6A-binding proteins^5^. With the advent of anti-m6A antibodies and methylated RNA immunoprecipitation sequencing techniques, significant progress has been made in the field of m6A in recent years, and the relationship between m6A modification and cancer is becoming clearer^6^. METTL3, an RNA methyltransferase, is associated with clinical prognosis in various carcinomas; however, whether it acts as an oncogene or a tumor suppressor depends on what gene is targeted in the carcinoma. We previously reported that pancreatic adenocarcinoma cell lines with low METTL3 expression are more sensitive to chemotherapeutic drugs, such as gemcitabine, 5-fluorouracil, and cisplatin, and radiotherapy; it provides a potential target for treating pancreatic adenocarcinoma^7^. However, the functional group of genes whose expression is directly regulated by METTL3 in pancreatic adenocarcinoma and the detailed mechanisms are still unknown.

Therefore, in this study, we performed a comprehensive analysis including m6A RNA immunoprecipitation (MeRIP) sequencing (MeRIP-seq) and site-specific methylation editing techniques to elucidate how METTL3 creates a favorable environment for pancreatic adenocarcinoma and to explore new therapeutic targets to improve the clinical outcomes in patients with pancreatic adenocarcinoma, which has a poor prognosis.

## Methods

### Cell lines and culture conditions

Human pancreatic cancer cell lines, MIAPaCa-2 and AsPC-1, were purchased from the American Type Culture Collection (Manassas, VA, USA). The cells were maintained in Dulbecco’s modified Eagle’s medium (DMEM) with a low level of glucose and RPMI1640 (Nacalai Tesque, Tokyo, Japan) supplemented with 10% fetal bovine serum (FBS) (Thermo-Fisher Scientific, Tokyo, Japan) and 1% penicillin/streptomycin (Life Technologies Japan, Tokyo, Japan) in 5% CO_2_ at 37 °C. METTL3-KD MIAPaCa-2 by short hairpin RNA was gifted by Kosuke Taketo^7^.

### Plasmid construction

The plasmid encoding dCas9 (pMJ841) was a gift from Jennifer Doudna (Addgene plasmid #39318)^8^. FTO plasmid was synthesized with optimized nucleotide sequence (GENEWIZ, Tokyo, Japan), and the sequence is shown in Supplementary Data 2. A dCas9–FTO plasmid was constructed according to a previous study^9^. In brief, pMJ841 of XbaI-site was removed by smoothing with Klenow fragment to XbaI resected fragment, and the stop codon of dCas9 was mutated to XbaI (PrimeSTAR® Mutagenesis Basal Kit, # R046A, Takara Bio, Shiga, Japan). Next, FTO was cloned downstream from dCas9 with a linker region coding for GGGGS using restriction enzymes XbaI and XhoI. For interference assay, we used pMXs–MCS6l–IN gifted by Hikichi et al.^10^. To construct the vectors for pMXs–dCas9–FTO, a fragment amplified using 5′-GCACTCGAGCACCATGGATAAGAAATACTCAA-3′ and 5′-CTAGCGGCCGCCTAGGGTTTTGCTTCCAGAA-3′ was inserted into XhoI–NotI sites of pMXs–MCS6l–IN. Similarly, pMXs–dCas9 was also constructed using primers of 5′-GCACTCGAGCACCATGGATAAGAAATACTCAA-3′ and 5′-CTAGCGGCCGCTTAGTCACCTCCTAGCTGAC-3′. All restriction enzymes were purchased from New England BioLabs Japan (Tokyo, Japan).

### Retrovirus preparation

To prepare retroviruses, platinum-E (Plat-E) cells^11^ were seeded at 1 × 10^6^ cells per 100-mm dish 1 day before transduction. Retroviral vector DNAs were introduced into Plat-E cells using Fugene HD transfection reagent (Promega, Tokyo, Japan) according to the manufacturer’s recommendations. The Fugene HD transfection reagent (18 μL) was diluted with 300-μL DMEM and plasmid DNA (6 μg) incubated for 15 min at room temperature. After incubation, the DNA/Fugene HD mixture was added dropwise onto Plat-E cells, which were then incubated overnight, and the medium was replaced 24 h later. After an additional 24 h, virus-containing supernatants derived from the Plat-E cultures were filtered with 0.22-µm pores (Merck Millipore, Burlington, MA, USA). Purified viral particles were infected to target MIA PaCa-2 cells with polybrene (Sigma-Aldrich, Tokyo, Japan). After a 24-h incubation, infected cells were selected with 400-µg/mL G418 (InvivoGen, Sandiego, CA, USA) for 14 days.

### MeRIP

MeRIP was conducted according to a previously reported protocol with minor modifications^12^. Briefly, we used ISOGEN (NIPPON GENE, Tokyo, Japan) to extract total RNA, and the extracted RNA was fragmented to 100 nt with ZnCl_2_ (FUJIFILM Wako Pure Chemical, Osaka, Japan) checked using an Agilent 2100 Bioanalyzer (Agilent technologies, Tokyo, Japan). Approximately 1/20 of the fragmented RNA was saved as the input control for further RNA sequencing. The remaining was incubated with an anti-m6A antibody (# 202 003, Synaptic Systems GmbH, Göttingen, Germany) at 4 °C for 2 h and then mixed with prewashed Pierce™ Protein A/G Magnetic Beads (# 10004D, Thermo-Fisher Scientific) in an immunoprecipitation buffer at 4 °C for 2 h. The mixture was obtained by magnetic separation. m6A containing RNA was eluted from the mixture using 6.7-mM N6-Methyladenosine 5-monophosphate sodium salt (# M2780, Sigma-Aldrich) at 4 °C for 2 h, and the methylated RNA was purified for further MeRIP-seq using RNeasy Mini kit (QIAGEN, Tokyo, Japan).

### MeRIP-seq

RNA libraries were constructed following manufacturer instructions using the TruSeq stranded mRNA Library Prep Kit (Illumina, USA) except for the process of using oligo-dT beads and sequenced by the HiSeq 2500 platform (Illumina). After checking the quality of sequencing using FastQC (https://www.bioinformatics.babraham.ac.uk/projects/fastqc/) and removing rRNA sequences using Bowtie2 (http://bowtie-bio.sourceforge.net/bowtie2/index.shtml)^13^, reads were aligned to human reference genomes and transcriptomes using HISAT2 (http://daehwankimlab.github.io/hisat2/)^14^. The human (hg19) reference sequences used for this purpose were downloaded from the University of California Santa Cruz Genome Browser. Peak analysis was performed using MACS2^15^ with some options: −q 0.01, –nomodel, and –extsize 100. We used input RNA sequencing data to perform expression analysis and calculated the reads using featureCounts (http://subread.sourceforge.net/)^16^. Read counting data were normalized and analyzed using DESeq2 (https://bioconductor.org/packages/release/bioc/html/DESeq2.html)^17^. The definition of significant peaks was fold change of more than 2 or less than − 2 and q value of less than 0.01, and the definition of differentially expressed genes was fold change log2 of more than 2 and *p* value of less than 0.01.

### Dataset comparison and network analysis

Differentially expressed genes whose log2 fold changes were more than 2 or less than − 2 in sh-METTL3 MIAPaCa-2 underwent GO functional enrichment analysis and protein–protein interaction network construction using Metascape (http://metascape.org/gp/index.html#/main/step1)^18^. We conducted pathway and process enrichment analysis using the following ontology sources—Kyoto Encyclopedia of Genes and Genomes Pathway^19^, Gene Ontology (GO) Biological Processes, Canonical Pathways^20^, Reactome Gene Sets^21^, and Comprehensive Resource of Mammalian Protein Complex^22^. Protein–protein interaction enrichment (PPI) analysis was performed using the BioGrid^23^, InWeb_IM^24^, and OmniPath^25^ databases. Network edges were visualized using Cytoscape (version 3.8.2; http://www.cytoscape.org/), a popular open source software^26^; subsequently, modules (highly interconnected regions) in a network were identified using MCODE, a Cytoscape plugin^27^, and dominator genes of each network were identified using MCDS^28^.

### GSEA analysis

The GSEA was performed using the MSigDB v7.5 collections—H, C2 and C5^29^. Metric for ranking genes were performed with “Signal2Noise” (in replicate samples) or “Ratio of Classes” (in no replicate samples). *p* Value < 0.05 and FDR (q-value) < 0.10 was considered significant for the GSEA analysis. Leading edge analysis was performed in significant “HALL MARK” gene sets. Other GSEA results not shown in the figure are listed in Supplementary data 3.

### Small interfering RNA (siRNA)

MIAPaCa-2 and AsPC-1 cells were seeded at 70–80% confluency in siRNA experiments. Target sequences of METTL3 were 5′-CCAGTCATAAACCAGATGA-3′ (almost same target sequences with MIAPaCa-2 sh-METTL3^7^) and siRNA (including negative control) were purchased from NIPPON GENE. They were transfected using Lipofectamine 3000 (Thermo-Fisher Scientific) as described in the standard protocol of Lipofectamine 3000. After 72 h of the transfection, total RNA and protein were extracted.

### Real-time quantitative reverse transcription PCR (qRT–PCR)

Total RNA was extracted from cultured cells using ISOGEN, and cDNA was synthesized using the PrimeScript™ RT Reagent Kit (# RR037A, Takara bio). qRT–PCR was performed with TB Green Premix Ex Taq II (# RR820B, Takara bio) using QuantStudio 5 (Applied Biosystems, Waltham, MA, USA). Furthermore, data were analyzed using the ΔΔCt method, in which target genes were normalized to glyceraldehyde-3-phosphate dehydrogenase. All measurements were performed in triplicate.

### MeRIP–qPCR

m6A modifications of *PLK1* were determined using MeRIP–qPCR assay^30^. qRT–PCR was performed using One-Step PrimeScript™ RT–PCR Kit (# RR064B, Takara Bio). Relative enrichment of m6A was normalized to the input: %Input = 1/20 × 2^Ct [IP]–Ct [input]^, and we calculated the ratio to the negative control.

### Transgenic mice experiments

Mice experiments were approved by the Osaka University Institute Animal Care and Use Committee following the principles and procedures outlined in the Japanese Act on the Welfare and Management of Animals and Guidelines for the Proper Conduct of Animal Experiments issued by the Scientific Council of Japan (number 24-122-039, approved on March 13, 2013, by Professor Y. Sawa). FVB/N-EL1-Luc/EL1-TAg mice (Common Name, EL1-Luc/EL1-TAg, abbreviated as EL1) were used to transgenic mice experiments, under the agreement (Caliper Life Science, Hopkinton, MA). Briefly, for co-injection to make EL1-Luc/EL1-TAg, two cDNA was used: the oncogenic cDNAs were prepared from 2.7-kb SV40 Large T-antigen, whereas the reporter cDNAs were prepared from Firefly Luciferase cDNA pGL3-Basic Vector (Promega). Each cDNA was fused with rodent pancreatic Elastase I promoter. For the construct of Mettl3 overexpressed transgenic mice, three 6-week-old female BL/6 mice were bled with two 6-week-old male BL/6 mice, and fertilized eggs were taken to perform cDNA injection in vitro to make transgenic animals. For the cDNA injection, the open-reading-flame of Mettl3 was cloned from C57BL/6 J mice by PCR, of which sequence were verified; Mettl3 cDNA was fused with chimeric CAG promoter, a composite of the cytomegalovirus (CMV) enhancer with the chicken beta actin promoter (CBA). The Mettl3 overexpressing mice were crossed with EL1 mice to make the double transgenic mice (WTg). Mice were confirmed as expected genotype, by PCR. The WTg mice formed abdominal tumors, which were apparent after 6 weeks; the luminous imaging followed to administration of vivoGlo Luciferin (SPI, Tokyo, Japan) as 150 µg/mouse(g) confirmed the presence of tumor(s) in pancreatic region under the IVIS imaging system (SPI), whereas the opened observation of abdomen indicated around 2-cm tumor(s), and tumors were processed for single-cell RNA sequencing (see below).

For the preparing murine primary cells cultured from tumors (MPC), we excited tumors from WTg mice, and tumor tissues were minced in DMEM with 10% FBS by utilizing 10-cm plastic culture dish. After removing debris, attached cells were maintained and passaged twice in sub-confluent condition. Independent formed colonies were isolated by PYREX cloning cylinders (Fisher Scientific, Waltham, MA), the MPC were cultured in the DMEM with 10% FBS. RNAs were extracted from cultured MPC by utilizing RNeasy mini kit for RNA extraction (Qiagen, Hilden, Germany), which were subjected to nucleotide sequencing. For the present study, we used the database of murine embryonal fibroblast (MEF) as the experimental control (GSE133753 and GSE193354).

### Preparation of single cells

To isolate single cells from surgically resected samples, pancreatic tumor tissue was washed with phosphate-buffered saline (PBS), cut into small pieces, and incubated with RPMI 1640 medium containing 10% FBS, 2 mg/mL of collagenase D (Roche, Basel, Switzerland) and 15 μg/mL of DNase I (Roche). The digested tissues were passed through a 40 μm cell strainer. The isolated cells were then washed in RPMI 1640 medium, incubated in 3 mL of ACK buffer for 3 min (to lysate red blood cells), and washed again in RPMI 1640 medium. Pancreatic tumor cells were collected in PBS containing 2% FBS. The isolated cells were stained with surface antibodies for 30 min at 4 °C, followed by 7AAD staining (BD Biosciences, Franklin Lakes, NJ). Flow cytometric analysis and cell sorting were performed using a FACSAria II instrument (BD Biosciences) to confirm the percentage of viable cells. Data were analyzed using FlowJo software (Tree Star, San Carlos, CA).

### Single-cell RNA sequencing (scRNAseq)

Single cell suspensions were processed with the 10× Genomics Chromium Controller according to the protocol described in the Chromium Single Cell 3′ Reagent Kits User Guide Chromium Next GEM Single Cell 3′ Kit v3.1 (Cat# PN-1000269), Chromium Next GEM Chip G Single Cell Kit (Cat# PN-1000127), and Dual Index Kit TT Set A (Cat# PN-1000215) were applied during processing. Following the manufacturer's recommendations, approximately 16,500 viable cells per sample were loaded into the Chromium controller to generate 10,000 single cell gel bead emulsions for library preparation and sequencing. Oil droplets of encapsulated single cells and barcoded beads (GEM) were then reverse transcribed on a Veriti Thermal Cycler (Thermo Fisher Scientific) to obtain cDNA tagged with cell barcodes and UMIs. The cDNA was then amplified and single-cell libraries were prepared according to the manufacturer's protocol. Quantification was performed using the Agilent Bioanalyzer High-Sensitivity DNA assay (Agilent, High-Sensitivity DNA Kit, Cat# 5067-4626). The amplified cDNA was then enzymatically fragmented, repaired at the ends, and polyA tagged. Amplified cDNA was cleaned/size-selected using SPRIselect magnetic beads (Beckman-Coulter, SPRIselect, Cat# B23317). Next, Illumina sequence adapters were ligated to the size-selected fragments and cleanup was performed using SPRIselect magnetic beads. Finally, sample indexes were selected, amplified, and double-sided size selection was performed using SPRIselect magnetic beads. Final library quality was assessed using the Agilent Bioanalyzer High-Sensitivity DNA assay. Samples were then sequenced in paired-end mode on a NovaSeq 6000 (Illumina) or DNBSEQ-G400RS (MGI) instrument. The resulting raw reads were processed on a cellranger (10× Genomics). scRNAseq data processing was performed with the R v4.1 and Seurat v4.0.5 packages.

### RIP assays

As previous report described^31^, RIP was performed with the Magna RIP RNA-Binding Protein Immunoprecipitation Kit (# 17–700, Sigma-Aldrich) according to the manufacturer’s instructions. Briefly, magnetic beads coated with 5-μg specific antibodies against mouse immunoglobulin G (#17–700, Sigma-Aldrich) or IGF2BP2 (ab#128175, Abcam, Tokyo, Japan) were incubated with prepared cell lysates overnight at 4 °C. Then, the RNA–protein complexes were washed 6 times and incubated with proteinase K digestion buffer. Finally, RNA was extracted using phenol–chloroform RNA extraction methods. The relative interaction between IGF2BP2 and the PLK1 3′UTR site was determined using qPCR and normalized to the input and MeRIP–qPCR.

### Western blotting

Western blotting was performed as our previous report^7^. Plated cells were carefully washed twice with PBS (Thermo-Fisher Scientific). Next, total protein was extracted using a radioimmunoprecipitation assay buffer (Thermo-Fisher Scientific) supplemented with 1% of Halt Protease Inhibitor Cocktail (100×) and Halt Phosphatase Inhibitor Cocktail (100×; Thermo-Fisher Scientific). NE-PER™ Nuclear and Cytoplasmic Extraction Reagents (# 78833, Thermo-Fisher Scientific) were used for protein extraction by separating from the nucleus and cytoplasm. Isolated proteins (10–30 µg/lane) were electrophoresed on 4–20% Mini-PROTEAN TGX Precast protein gels (Bio-Rad Laboratories, Tokyo, Japan) and transferred to an Immobilon-P polyvinylidene fluoride membrane (Sigma-Aldrich) using Mini Trans-Blot® Cell (Bio-Rad Laboratories). In order to perform quickly the expression analysis of high and low molecular weight protein on the same membrane, the membrane was cut and analyzed at any level from 40 to 90 kDa. The membrane was blocked with 5% skim milk for 1 h at room temperature and reacted with appropriate dilutions of primary antibody overnight at 4 °C. The membrane was washed thrice with Tris-buffered saline-Tween (TBST) for 5 min each and incubated with horseradish peroxidase-linked anti-rabbit immunoglobulin G (GE Healthcare Biosciences, Tokyo, Japan) for 1 h at room temperature. The membrane was washed again with TBST thrice for 5 min each. Next, the secondary antibodies were identified using Clarity Western ECL substrate (Bio-Rad Laboratories), a detection reagent. Images were acquired using ChemiDoc™ Touch (Bio-Rad Laboratories). The antibodies used were as follows: METTL3: #ab195352 (Abcam), PLK1: #4513 (Cell Signaling Technology, Tokyo, Japan), ACTB: #4967 (Cell Signaling Technology), phospho-H3: #sc-374669 (Santa Cruz, Dallas, TX, USA), CCNB1: #sc-245 (Santa Cruz), phospho-ATM (S1981): #13050 (Cell Signaling Technology), phospho-CHEK2 (T68): #2197 (Cell Signaling Technology), phospho-ATR #2853 (S428): (Cell Signaling Technology), phospho-CHEK1 (S345): #2438 (Cell Signaling Technology), phospho-H2AX (S139): #9178 (Cell Signaling Technology), H3: #4499 (Cell Signaling Technology), Cas9: #632607 (Takara Bio), FTO: #14386 (Cell Signaling Technology), ATR: #sc-515173 (Santa Cruz), RM2: #sc-398294 (Santa Cruz), RPA2:#sc-56770 (Santa Cruz), phospho-RPA2 (S33): #A300-246A-M (BETHYL, Montgomery, TX, USA).

### Immunohistochemical staining

Immunohistochemical staining was performed using a previously described method with minor modifications^32^. Briefly, paraffin blocks were cut into 4-µm sections. Next, the sections were deparaffinized in Hemo-De (Falma, Tokyo, Japan) and treated with Liberate Antibody Binding Solution (Polysciences, Warrington, PA, USA); endogenous peroxidase activity was blocked using 1% hydrogen peroxide for 30 min at 4 °C, and the sections were incubated overnight at 4 °C with a specific antibody (anti-PLK1 antibody: mouse monoclonal, 1:500 dilution [# ab17056, Abcam], and anti-METTL3 antibody: rabbit monoclonal, 1:1000 dilution [# ab195352, Abcam]). After that, the sections were incubated with a secondary antibody for 1 h at room temperature and assessed using avidin–biotin complex reagents (Vector Laboratories, Burlingame, CA, USA). The sections stained for PLK1 and METTL3 were incubated in DAB for 8 min and 17 min, respectively. Subsequently, all sections were counterstained with hematoxylin. To analyze the relative expression level of PLK1 and METTL3, we used Color Deconvolution (https://github.com/fiji/Colour_Deconvolution), a plugin for ImageJ (https://imagej.nih.gov/ij/)^33^. Briefly, the part of DAB separated using Color Deconvolution was binarized with a certain threshold, and the concentration intensities were measured at three locations in a certain area, and the average was compared among each sample. The use of paraffin blocks in this study was approved by the Osaka University Institutional Review Board (approval number. 15149-2, 19439). We obtained written consent from all patients at the beginning of the study. This study was conducted as a retrospective study of samples.

### Flow cytometric analysis

In the cell cycle analysis, cells were fixed with ice-cold 70% ethanol, resuspended, and incubated in PBS containing propidium iodide (50 µg/mL) at room temperature for 20 min after treatment with RNase (100 µg/mL). Expression analyses were performed according to the manufacturer’s instructions (eBioscience™ Foxp3/Transcription Factor Staining Buffer Set; # 00-5523-00, Thermo-Fisher Scientific). Briefly, the cells after fixation and permeabilization were incubated with anti-PLK1 antibody (# ab17056, Abcam) and anti-METTL3 antibody (# ab195352, Abcam) at 4 °C for 1 h. Then, the secondary antibodies (Goat-anti-Rabbit Alexa 488: #A1008 and Goat-anti-Mouse Alexa647: #A21235, Thermo-Fisher Scientific) were incubated at room temperature for 30 min, and nuclear staining was performed using DAPI (NucBlue™ Fixed Cell Stain, Thermo-Fisher Scientific). The stained cells were analyzed using the BD FACS Canto II flow cytometer (BD Biosciences, Tokyo, Japan) and FlowJo (BD Biosciences). Over 50,000 cells were analyzed per sample.

### Clonogenic assay

Clonogenic assay was performed as previously described^34^. Cells (0.5–30 × 10^3^) were plated in triplicate into 60-mm dishes and pretreated with NMS-P937 for 24 h. After this, the cells were irradiated with doses of 0, 2, 4, 6, and 8 Gy. The cells were irradiated with Gammacell® 40 Exactor (MDS Nordion, Ontario, Canada), a ^137^Cs gamma-ray therapeutic irradiator, with a dose rate of 0.85 Gy/min, at Osaka University Graduate School of Medicine. Thirty minutes after irradiation, the cells were washed with PBS, and fresh medium was added to the dish. After 10–14 days, cell colonies (more than 50 cells) were stained with crystal violet and counted using an automatic colony counter (PSF-2000; Shashin Kagaku, Kyoto, Japan). Surviving fractions were calculated and normalized to the nonirradiated controls for each group. We performed the statistical analysis, ANOVA based tests, using the R package “CFAssay”^35^.

### Immunocytochemistry

In this study, cells were seeded into 6-well dishes with cover glasses and incubated. Then, these cells were fixed with 4% paraformaldehyde/PBS at 37 °C for 15 min and permeated with PBS containing 0.5% Triton X-100 at room temperature for 15 min. Next, the cells were blocked in a blocking buffer (PBS containing 3% bovine serum albumin; Sigma-Aldrich) at room temperature for 1 h. Afterward, the cells were incubated with anti-PLK1 antibody (#ab17056, Abcam) and anti-METTL3 antibody (#ab195352, Abcam) or a-Tubulin (#sc-5286, Santa Cruz) as the primary antibody at room temperature for 1 h. Then, the cells were incubated with Goat-anti-Rabbit Alexa 488 (#A1008, Thermo-Fisher Scientific) and Goat-anti-Mouse Alexa647 (#A21235, Thermo-Fisher Scientific) as secondary antibodies at room temperature for 30 min. Furthermore, nuclei were stained with DAPI (NucBlue™ Fixed Cell Stain, Thermo-Fisher Scientific). Finally, images were captured using a BZ-X800 microscope (Keyence, Osaka, Japan). The fluorescence intensities of PLK1, METTL3, and DAPI were measured using a BZ-X Analyzer (Keyence), and 20 cells in the mitotic phase and 20 cells in the interphase were randomly selected for comparison. In mitotic catastrophe assay, similarly, 200 cells were randomly extracted, and the number of nuclei per cell was measured.

### Cell sorting using FUCCI

HCT-116, which stably expressed fluorescent ubiquitination-based cell cycle indicator (FUCCI)-green (mAG) and FUCCI-red (mKO2) established by Miyawaki et al.^36^, was prepared as described by a gift from Kagawa et al.^37^. To analyze the gene expression levels of G1 and G2/M, cells were selected by single-cell sorting using a Sony SH800 cell sorter (Sony, Tokyo, Japan). mAG and mKO2 were excited using 488-nm and 561-nm lasers, respectively, and their emission was detected using 530/30BP and 585/42BP filters, respectively^38^. Total RNA was extracted from the sorted cells with ISOGEN (Nippon gene).

### sgRNA and PAMmer synthesis and cell transfection for RNA m6A editing

PAMmer of the target region to edit site-specific m6A was selected according to previous reports^9,39^. Briefly, the PAM sequence of PAMmer was set 19 bases downstream of the target m6A site, and the sgRNA corresponding to the site was synthesized using Guide-it™ sgRNA In Vitro Transcription (Takara Bio). A PAMmer was composed of mixed 2′OMe RNA and DNA bases and purified by HPLC (Ajinomoto Bio-Pharma, Tokyo, Japan). Complexes of sgRNA and PAMmer were transfected into the cells with Lipofectamine 3000 (Thermo-Fisher Scientific) according to the instructional protocols^40,41^. In 100-mm dishes, 150-pmol sgRNA and 150-pmol PAMmer were dissolved in Lipofectamine 3000 and Opti-Mem (Thermo-Fisher Scientific) without P3000 Reagent and administered to the cells. After 48 h, the cells were used for various experiments.

### Annexin V assays

Apoptosis assay was performed using APC Annexin V Apoptosis Detection Kit with 7-AAD (BioLegend, Sandiego, CA, USA) according to the instructional protocols. The fluorescence intensity of Annexin V-APC and 7-AAD was measured after gating viable cells on FSC vs. SSC dot plots using the BD FACS Canto II system (BD Biosciences).

### Cell counting kit-8 (CCK-8) assay

The indicated drugs were administered 1 day after 1000 cells were spread in 96-well plates, and cell proliferation was assessed using CCK-8 assay (Dojindo, Kumamoto, Japan) 72 h after treatment following the manufacturer’s instructions. Optical density was recorded at 450 nm using a Luminoskan™ Microplate Luminometer (Thermo-Fisher Scientific). The combination index was calculated as in previous reports^42,43^.

### Clinical samples

Tissue samples of pancreatic adenocarcinoma were obtained during surgery. All patients had undergone preoperative radiation therapy and resection of the primary tumor at Osaka University Hospital (Osaka, Japan), and had been under observation for at least 2 years postoperatively. Informed consent was obtained from all patients. We have complied with all relevant ethical codes for work on human subjects. The study protocol was approved by the Ethics Committee of Osaka University (Approval No.: 19439).

### Statistical analysis

The results are expressed as the mean ± standard deviation (SD). Data were analyzed using Student’s t-test to determine statistically significant changes. OS and RFS were calculated as the time from the start of CCRT to death from any cause and relapse, respectively. The time to relapse of local area was calculated as the time from the start of CCRT to the recurrence of pancreatic adenocarcinoma in the field of irradiation, except for any death without relapse, which was regarded as competing risks. The probabilities of OS and RFS were estimated using the Kaplan–Meier method, and PLK1 expression levels were compared using the log-rank test. The local relapse rate was estimated based on a cumulative incidence, and the results were compared using Gray’s test. All tests were two-sided, and *p* values of less than 0.05 were used to denote statistical significance. All statistical analyses were performed using EZR (version 1.42; Saitama Medical Center, Jichi Medical University, Saitama, Japan)^44^, which is a graphical user interface for R 4.0.0 (The R Foundation for Statistical Computing, Austria). The graphs were drawn using R and Prism 9 (GraphPad Software, Sandiego, CA, USA).

## Results

### METTL3 upregulates PLK1 by methylating 3′UTR in pancreatic adenocarcinoma cell line

**Figure 1:**
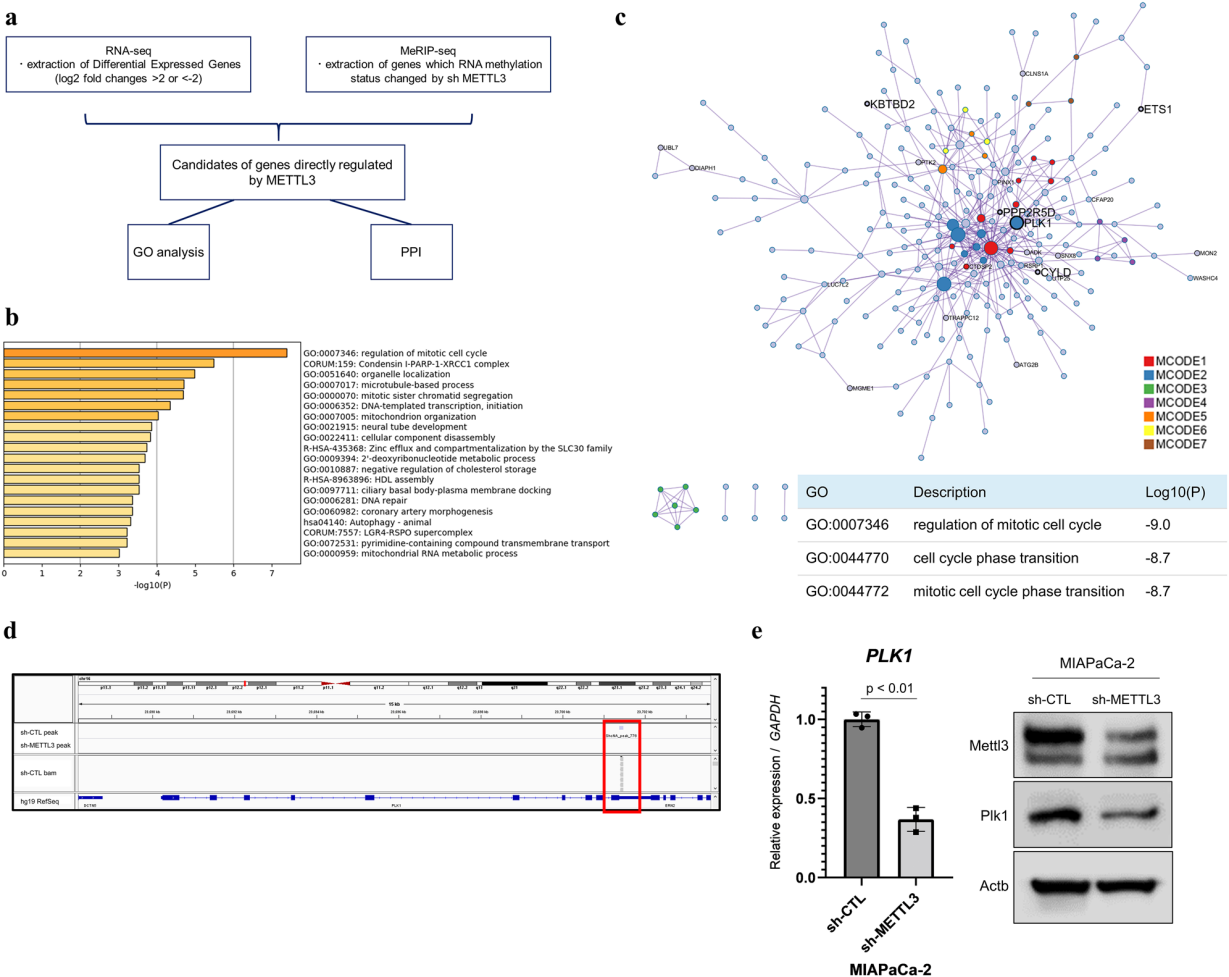
MeRIP-seq and network analysis to explore the targets of METTL3. (**a**) Schema of MeRIP-seq and RNA expression analysis. Differential expressed genes (fold change log2 of more than 2 or less than − 2 and *p* value of less than 0.01) in control cells compared with METTL3-KD cells, and genes with altered methylation status due to METTL3 KD are extracted as the candidates of genes directly regulated by METTL3. (**b**) Pathway and process enrichment analysis using the 553 identified genes upregulated by METTL3. The ontology term of “regulation of mitotic cell cycle” is the most statistically significant term among the gene clusters (Log10(P) = − 7.39; Log10(q) = − 3.07). (**c**) PPI analysis in a group of genes regulated by METTL3. “Regulation of mitotic cell cycle,” “cell cycle phase transition,” and “mitotic cell cycle phase transition” are identified as the three most statistically significant GO terms. The names of the genes whose methylation peaks are lost due to METTL3 KD are listed, and those whose methylation peaks in the 3′UTR are lost are listed in larger letters. Among them, PLK1 is selected as the most important hub gene in the network by MCDS analysis. (**d**) Loss of methylation peak in the PLK1 3′UTR due to METTL3 KD. (**e**) Relative expression changes of PLK1 mRNA and protein along with decreased METTL3 expression. Values of qPCR are the mean ± SD of 3 in independent experiments. Two-tailed Student’s t-tests were used. Uncropped images are shown in Supplementary Fig. 7.

We performed MeRIP-seq of METTL3-knockdown (KD) MIAPaCa-2 and control cells to detect METTL3-methylated genes and extracted genes whose methylation was lost in METTL3-KD cells compared with control cells. Next, we performed mRNA expression analysis using dates of input samples at MeRIP-seq (Fig. 1a). As previously reported, mRNA methylation is predominantly found in the 3′ untranslated region (UTR)^45^, and in this study, the methylation of mRNA was predominantly found in the 3′UTR in the significant peaks of MeRIP-seq (Supplementary Fig. 1a). Furthermore, our previous study has revealed that METTL3 acts on chemotherapy and radiotherapy resistance in pancreatic cancer cell lines^7^. To search for new targets of therapeutic resistance, we extracted a group of genes whose expression was upregulated in control cells compared with METTL3-KD cells in the expression comparison analysis. We conducted pathway and process enrichment analysis for the 553 genes identified to be upregulated by METTL3 and revealed that “regulation of mitotic cell cycle” was the most statistically significant term among the gene clusters (Log10(P) = − 7.39, Log10(q) = − 3.07) (Fig. 1b). Next, PPI analysis provided 246 nodes (genes) and 410 edges (interactions) in the network (Fig. 1c), and additional analysis identified seven modules in MCODE (Supplementary Fig. 1b). Moreover, in the PPI analysis, “regulation of mitotic cell cycle,” “cell cycle phase transition,” and “mitotic cell cycle phase transition” were identified as the three most statistically significant GO terms. After extracting genes whose methylation is regulated by METTL3, we identified polo-like kinase 1 (PLK1) as the most important hub gene in the network by MCDS analysis. We confirmed a loss of the methylation peak of PLK1 3′UTR using the Integrative Genomics Viewer^46^ in METTL3-KD cells (Fig. 1d), and the predicted score based on the nucleotide sequence of the methylation site was high in sequence-based RNA adenosine methylation site predictor^47^, indicating a plausible methylation site (Supplementary Fig. 1c). Then, we confirmed the reduction in the expression level of mRNA and protein in METTL3-KD cells compared with that in control cells (Fig. 1e). Furthermore, we performed siRNA experiments to confirm whether PLK1 expression is regulated by METTL3 in another pancreatic cancer cell line (AsPC-1), and confirmed that PLK1 expression is downregulated by METTL3-KD in AsPC-1 as in MIAPaCa-2 (Supplementary Fig. 1d,e).These findings indicate that PLK1 is an important gene regulated via RNA methylation by METTL3, suggesting that it is responsible for various treatment resistances in pancreatic adenocarcinoma because we revealed that control cells were more resistant to chemotherapy and radiotherapy than METTL3-KD cells^7^.

### High PLK1 expression is an important prognostic risk factor in patients with pancreatic adenocarcinoma

**Figure 2:**
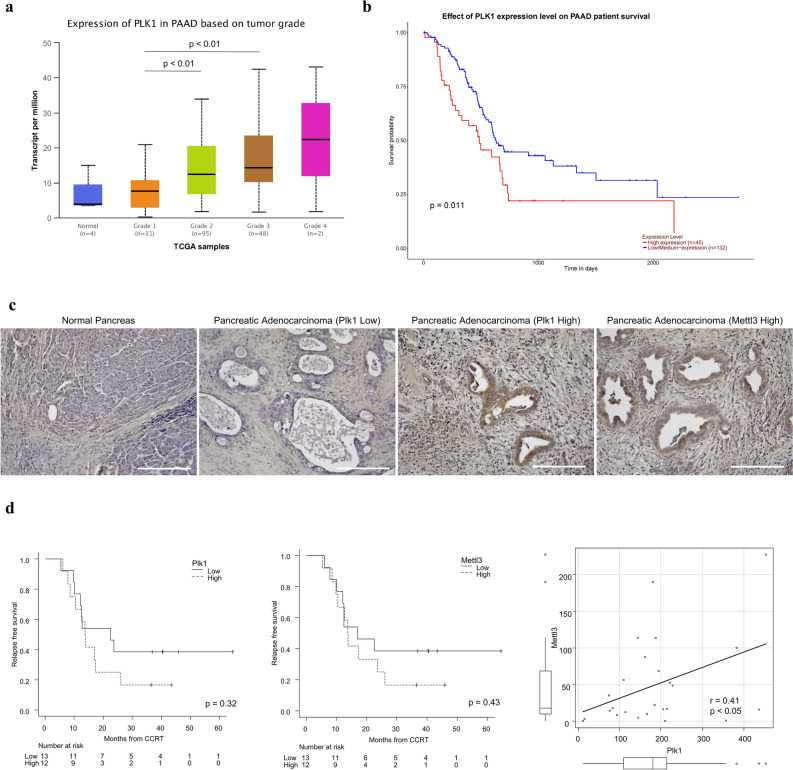
PLK1 is a poor prognostic factor for pancreatic adenocarcinoma. (**a**,**b**) Clinical impact of PLK1 from TCGA data via UALCAN (http://ualcan.path.uab.edu/). (**a**) PLK1 is elevated in parallel with the pathological grade of pancreatic adenocarcinoma. (**b**) Patients with pancreatic cancer with elevated PLK1 have poor OS. (**c**) Histopathological images of PLK1 in patients with pancreatic adenocarcinoma (immunohistochemistry with DAB). PLK1 expression is low in healthy pancreatic tissue, whereas PLK1 is abundantly expressed in pancreatic adenocarcinoma tissue. Pancreatic adenocarcinoma with high METTL3 expression is also presented (same patient with high PLK1). The scale represents 200 µm. (**d**) High protein expression of PLK1 and METTL3 tends to be associated with poor prognosis in pancreatic adenocarcinoma. A positive correlation between PLK1 and METTL3 was also observed.

Next, we investigated whether high PLK1 expression is a prognostic factor for pancreatic adenocarcinoma using public databases. In genomics data from The Cancer Genome Atlas (TCGA) via UALCAN (http://ualcan.path.uab.edu/)^48^, PLK1 expression increases with the elevation of pancreatic adenocarcinoma histological grade, and high PLK1 expression is a significant factor for poor prognosis in the overall survival (OS) of patients with pancreatic adenocarcinoma (Fig. 2a,b). Furthermore, PLK1 has few mutations (0.5%) compared with other genes (i.e., TP53, KRAS, CDKN2A, and SMAD4) that are frequently mutated in pancreatic adenocarcinoma in cBioPortal (https://www.cbioportal.org/)^49,50^, and the promoter methylation level did not differ from that in healthy pancreatic tissues. This result suggests that the effect of PLK1 expression changes due to RNA modification is greater than that of DNA modifications, including mutations (Supplementary Fig. 2a,b). In addition to the mRNA expression level of PLK1, we examined its protein expression level. It was revealed that the protein expression level of PLK1 was higher in pancreatic adenocarcinoma than that in healthy pancreas from the Human Protein Atlas (https://www.proteinatlas.org/)^51^; however, its prognostic contribution was unclear (Supplementary Fig. 2c). Therefore, we performed immunohistochemical staining of pancreatic adenocarcinoma specimens from patients subjected to preoperative concurrent chemoradiotherapy (CCRT) to investigate the relationship between PLK1 protein expression level and prognosis. The protein expression level of PLK1 was quantitatively analyzed after extracting the 3,3′-diaminobenzidine (DAB) component and binarizing it as in Supplementary Fig. 2d. Samples of healthy pancreatic tissue and pancreatic adenocarcinoma specimens with low and high PLK1 expression levels are shown in Fig. 2c. We analyzed 25 patients and did not find a correlation between PLK1 and overall response to preoperative CCRT (data not shown). Although the number of cases was small and no statistically significant difference was observed, high PLK1 protein expression level tended to be a poor prognostic factor for relapse-free survival (Fig. 2d). In addition to PLK1, we also evaluated the protein expression level of METTL3; similar to PLK1, high METTL3 expression tended to be a risk factor for relapse-free survival. A weak but significant positive correlation between PLK1 and METTL3 was also observed (Fig. 2d). These results indicate that high PLK1 expression may be an adverse prognostic factor in patients with pancreatic adenocarcinoma.

### PLK1 inhibition using NMS-P937, a selective small molecule PLK1 inhibitor, increases radiosensitivity via G2/M arrest

**Figure 3:**
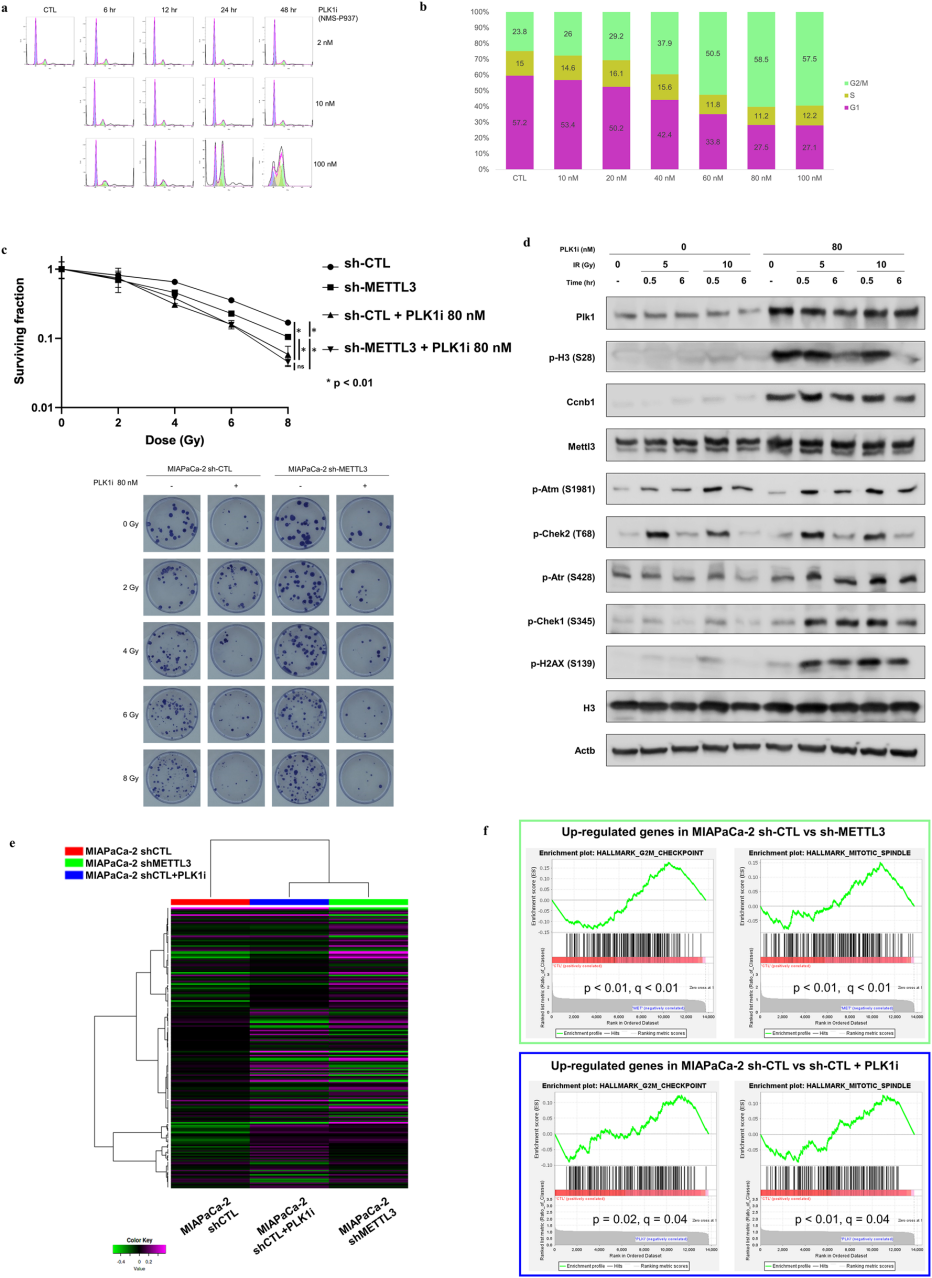
PLK1 inhibition increases radiosensitivity via G2/M arrest. (**a**,**b**) Cell cycle analysis with NMS-P937, a PLK1 inhibitor, which induces G2/M arrest in a time- and concentration-dependent manner. The optimal concentration for the most effective induction of G2/M arrest is 80 nM for 24 h. (**c**) A clonogenic assay to evaluate the synergistic effect of NMS-P937. The addition of NMS-P937 caused more synergistic effects with irradiation, regardless of the intensity of METTL3 expression. Values are the mean ± SD of 3 and the *p* value in *p < 0.01 was determined by ANOVA based tests using R package “CFAssay”. (**d**) Western blotting for identifying the mechanism of enhanced radiosensitivity of NMS-P937. Radiosensitivity by PLK1 inhibition is due to apoptosis introduced by the ATR–CHEK1 pathway as a result of DNA double-strand breaks increased by G2/M arrest. Uncropped images are shown in Supplementary Fig. 7. (**e**) Heatmap clustered based on similarity of expression profiles. MIAPaCa-2 sh-CTL approached sh-METTL3 in expression profile with the addition of PLK1i. (**f**) GSEA of down-regulated genes by METTL3-KD and PLK1i identified a group of genes involved in the mitosis. *PLK1i means NMS-P937 in this figure.

We investigated the effect of PLK1 inhibition on pancreatic cancer cells using NMS-P937, a selective PLK1 inhibitor (PLK1i), which has 5000-fold higher selectivity than PLK2/PLK3^52^ (Supplementary Fig. 3a). NMS-P937 has been reported to be effective mainly against hematological cancers; however, it was also effective against pancreatic cancer cells, with a half-maximal inhibitory concentration (IC50) of 23.5 nM for MIAPaCa-2 (Supplementary Fig. 3b). Since PLK1 inhibition exerts its antitumor activity by inducing G2/M cell cycle block^52^, we first performed a concentration- and time-dependent cell cycle assay to determine the optimal concentration of NMS-P937 for inducing G2/M arrest in MIAPaCa-2 (Fig. 3a). We considered that exposure to the drug for more than 48 h was too high cytotoxicity for evaluating synergistic effects, and we evaluated it by shifting the concentration to 10–100 nM for 24 h of exposure (Fig. 3b). As a result, we defined the optimal condition to induce G2/M arrest as 80 nM for 24 h. Then, we performed a clonogenic assay to evaluate the synergistic effect of NMS-P937. Although we did not find any synergistic effect with the addition of 40-nM NMS-P937, 80-nM NMS-P937, the concentration that induces G2/M arrest, had a synergistic effect (Supplementary Fig. 3c). In the analysis of METTL3-KD and control cells, METTL3 depletion enhanced radiosensitivity as previously reported^7^, and the addition of NMS-P937, PLK1i, had synergistic effects with irradiation, regardless of the intensity of METTL3 expression (Fig. 3c), indicating that PLK1 inhibition spares the radioresistance due to METTL3 elevation.

To elucidate the mechanism of synergy between PLK1i and irradiation, we analyzed various pathways using Western blotting (Fig. 3d). After 24 h of PLK1i exposure and the same amount of dimethyl sulfoxide (DMSO) as a control, the cells were irradiated at 5 Gy and 10 Gy, and the total proteins were collected 30 min and 6 h after irradiation, respectively. The increased expression of PLK1 following treatment could be consequent to the attempted override of drug-induced PLK1 inhibition through a feedback mechanism of expression of higher amounts of protein. The increased phosphorylation of the well-known mitotic markers histone H3 on Ser28 and cyclin B1 further confirms the mitotic block obtained with the inhibitor and thus effective PLK1 inhibition. The upregulation of METTL3 expression by radiotherapy in the DMSO-treated group may be related to the involvement of METTL3 in DNA damage response^53^. Interestingly, METTL3 expression was also upregulated by G2/M phase quiescence induced by PLK1 inhibition with or without irradiation. This result suggests the cell cycle-dependent expression changes in METTL3. Although the activation of the ataxia telangiectasia mutated (ATM)–checkpoint kinase 2 (CHEK2) pathway was predominant only after irradiation treatment in combination with NMS-P937, DNA double-strand breaks (DSBs) were elevated as indicated by the increased phosphorylation of H2A.X variant histone (H2AX), which is required for checkpoint-mediated arrest of cell cycle progression in response to low doses of ionizing radiation and for efficient repair of DNA DSBs specifically by the phosphorylation of C-terminal region^54^ whereas the ataxia telangiectasia and Rad3-related (ATR)–CHEK1 pathway was activated^55^, indicating the possibility that the enhancement of radiosensitivity by PLK1 inhibition is due to apoptosis introduced by the ATR–CHEK1 pathway due to DNA double-strand breaks increased by G2/M arrest.

Next, we evaluated the changes in gene expression paterns induced by PLK1 inhibition and METTL3-KD: total RNA was extracted from three cell types, MIAPaCa-2 sh-CTL, MIAPaCa-2 sh-METTL3, as well as MIAPaCa-2 sh-CTL treated with PLK1i, and RNA- sequencing was performed. Clustering by heatmap showed that the addition of PLK1i resulted in a similar expression pattern to that of sh-METTL3 (Fig. 3e), and GO/PPI analysis of genes commonly down-regulated by METTL3-KD and PLK1i revealed a group of genes involved in the cell cycle (Supplementary Fig. 3d–f). GSEA analysis of sh-CTL cells with sh-METTL3 or PLK1i addition showed that the gene set related to mitosis was enriched in both conditions (Fig. 3f). These results suggest that in pancreatic cancer cells, elevated METTL3 expression mainly regulates genes involved in the cell cycle, and that PLK1 inhibition may have a similar effect to decreased METTL3 expression.

### Elevated METTL3 expression affects the cell cycle and tumor microenvironment in a murine model of pancreatic carcinogenesis

**Figure 4:**
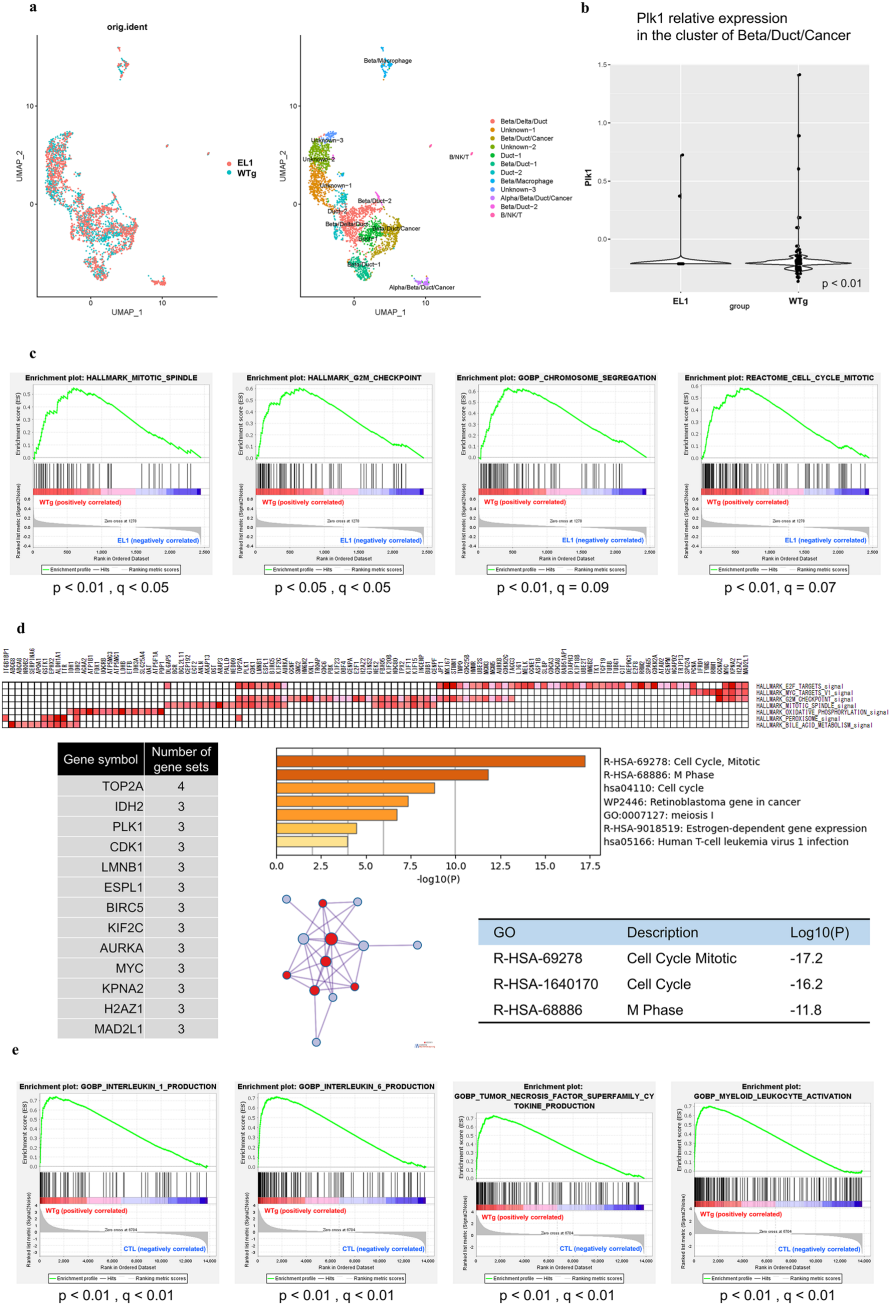
scRNA-seq and bulk RNA-seq of pancreatic tumors in a WTg mouse model overexpressing METTL3. (**a**) The data was summarized in the UMAP and color-coded according to sample type (left), and cell type (right). (**b**) The violin plot shows the comparison of PLK1 expression between WTg and EL1 in the cluster of Beta/Duct/Cancer. (**c**) GSEA shows the gene sets of cell cycle and mitosis in the cluster of Beta/Duct/Cancer were significantly enriched in WTg compared with EL1. (**d**) The leading edge analysis shows enriched genes in the cluster of Beta/Duct/Cancer. GO/PPI analysis is shown for genes that appeared in the “HALLMARK” gene sets three or more times. (**e**) GSEA shows enriched gene sets of nine clones of murine primary cells cultured from tumors of WTg mice compared with six types of mouse embryonic fibroblasts obtained from GSE133753 and GSE193354.

We tested how elevated METTL3 expression affects pancreatic cancer in a mouse model. We performed single-cell RNA-sequencing of pancreatic cancer tissue from control (EL1) and METTL3-overexpressing (WTg) mice and bulk RNA-sequencing using murine primary cells cultured from tumors (MPC) derived from pancreatic cancer tissue (Supplementary Fig. 4a,b). The data of single-cell RNA-sequencing included 12 cell types with reasonably differentially expressed genes (Fig. 4a, Supplementary data 4 and Supplementary Fig. 4c,d). Among these cell populations, we analyzed the clusters overexpressing METTL3 and recognized as cancer (Beta/Duct/Cancer) and the surrounding clusters recognized as normal pancreatic ducts (Duct-1, Beta/Duct-1) (Supplementary Fig. 4e). First, we confirmed increased expression of PLK1 in the Beta/Duct/Cancer cluster due to increased METTL3 expression (Fig. 4b). Next, GSEA analysis of each cluster showed that gene sets related to cell cycle and mitosis were significantly enriched in the WTg group in the Beta/Duct/Cancer cluster (Fig. 4c). In the genes extracted from the leading edge analysis, GO/PPI analysis showed that many genes related to the cell cycle were enriched in gene sets (Fig. 4d). On the other hand, in the cluster of normal pancreatic ducts (Duct-1, Beta/Duct-1), these genes related to the cell cycle were not significantly enriched (Supplementary Fig. 4f,g).

Nine clones of MPC from WTg mice could be established, but not from EL-1. Six types of MEFs from public data were used as controls because the MPCs were morphologically and empirically fibroblasts. The expression quantification and GSEA analysis of each MEF showed that a group of genes involved in cytokine production and inflammation, such as IL-1, IL-6, and TNFα, were enriched in the WTg group (Fig. 4e), and these cytokines have been reported to induce a favorable environment for tumors in general^56^. In a murine model of pancreatic carcinogenesis, these results suggest that elevated METTL3 expression affects the cell cycle including PLK1 expression in pancreatic cancer cells and may also be involved in the pathogenesis of pancreatic cancer by creating a favorable environment for surrounding fibroblasts for the tumor.

### METTL3 is regulated in a cell cycle-dependent manner

**Figure 5:**
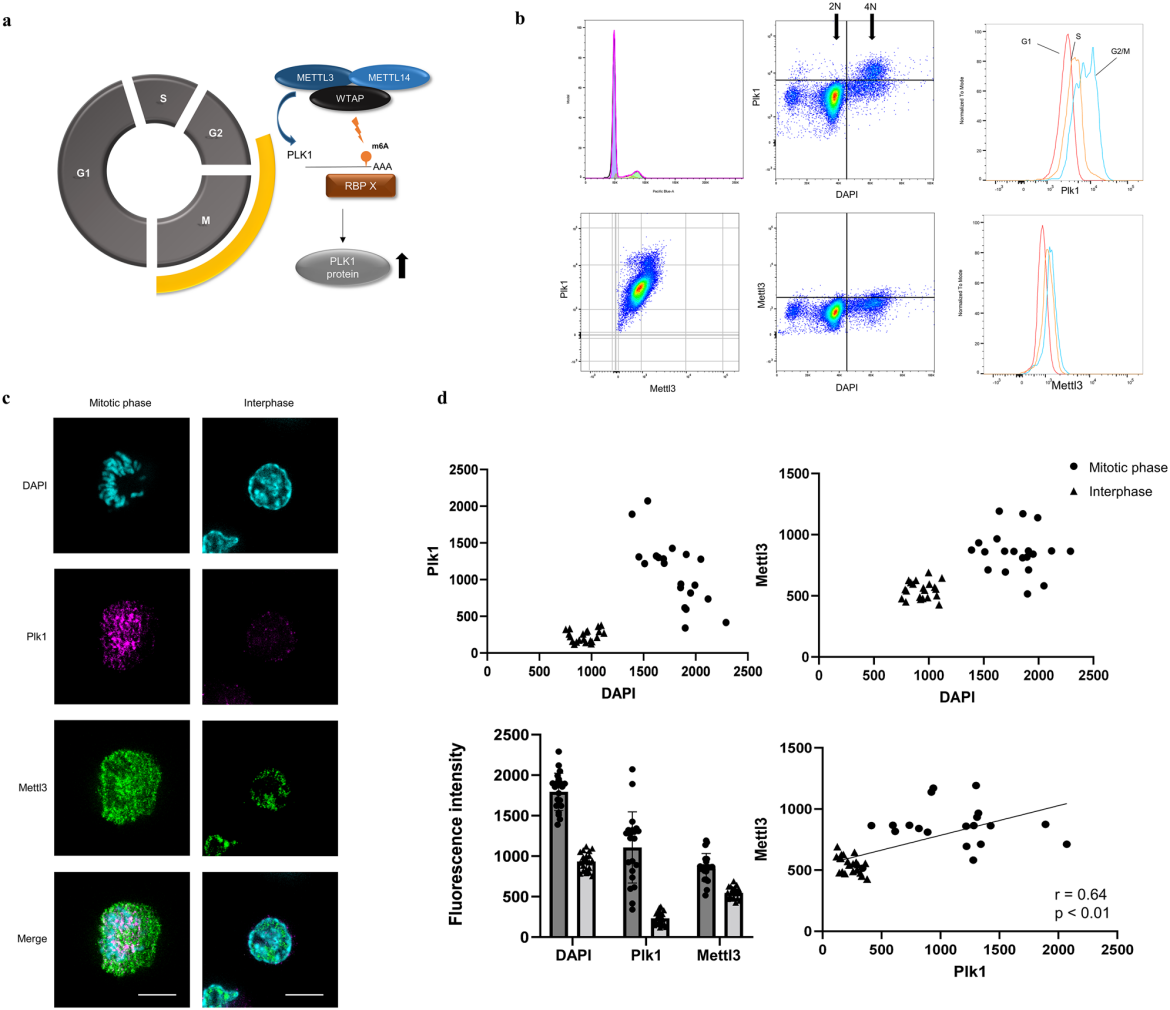
METTL3 is expressed in a cell cycle-dependent manner similar to PLK1. (**a**) Is METTL3 regulated in a cell cycle-dependent manner? We hypothesized that METTL3 regulates cell cycle-dependent changes in PLK1 expression by methylating the 3′UTR of PLK1. (**b**) Flow cytometric analysis to observe the expression changes of METTL3 and PLK1 during the cell cycle. PLK1 is upregulated in the G2/M phase (upper middle and upper right). Similarly, the expression of METTL3 was slightly upregulated by 4 folds of that in DNA (lower middle) and G2/M phase (lower right), and the positive correlation with PLK1 expression was observed (lower left). (**c**,**d**) The expression of METTL3 (green) and PLK1 (magenta) was compared between the M phase and interphase using immunocytochemistry. The scale represents 20 µm. The fluorescence intensity of DAPI doubled in the M phase reflecting the amount of DNA, and the fluorescence intensity of PLK1 and METTL3 increased accordingly. Furthermore, a correlation was found between the fluorescence intensities of PLK1 and METTL3 (Pearson correlation, r = 0.64, *p* < 0.01).

We considered that METTL3 regulated the expression of PLK1 in a cell cycle-dependent manner. Specifically, we hypothesized that METTL3 expression increases from the late G2 phase to the M phase when PLK1 expression is upregulated^57^, increasing the methylation of the PLK1 3′UTR, which in turn increases mRNA stability through the action of some RNA-binding proteins, leading to increased PLK1 protein expression (Fig. 5a). First, we performed flow cytometric analysis to observe the expression changes of METTL3 and PLK1 during the cell cycle (Fig. 5b). PLK1 was upregulated by 4 folds of that in DNA, as previously reported. Cell cycle analysis and comparison of normalized fluorescence intensity for each cycle phase also showed that PLK1 was upregulated in the G2/M phase. Similarly, the expression of METTL3 was slightly upregulated by 4 folds of that in DNA and the G2/M phase, and a positive correlation with PLK1 expression was observed. In addition to flow cytometric analysis, we confirmed the expression change of METTL3 in a cell cycle-dependent manner using immunocytochemistry. The mitotic phase was identified by 4′,6-diamidino-2-phenylindole (DAPI) staining (Fig. 5c), and we compared the fluorescence intensities of PLK1 (magenta) and METTL3 (green) between the mitotic phase and interphase after randomly selecting 20 cells. The DAPI of the mitotic phase and interphase was approximately doubled, reflecting the amount of DNA, and the expressions of METTL3 and PLK1 were increased in the mitotic phase compared with those in the interphase. Similar to the results of the flow cytometric analysis, a positive correlation between METTL3 and PLK1 was observed (Fig. 5d). A similar expression variation in mRNA was also confirmed in the other cell line (HCT-116) using fluorescent ubiquitination-based cell cycle indicator (FUCCI) (Supplementary Fig. 5a). These results indicated that METTL3 and PLK1 are upregulated in the mitotic phase and the possibility that METTL3 is involved in the cell cycle-dependent upregulation of PLK1.

### Site-specific RNA N6-methyladenosine demethylation by dCas9-FTO

**Figure 6:**
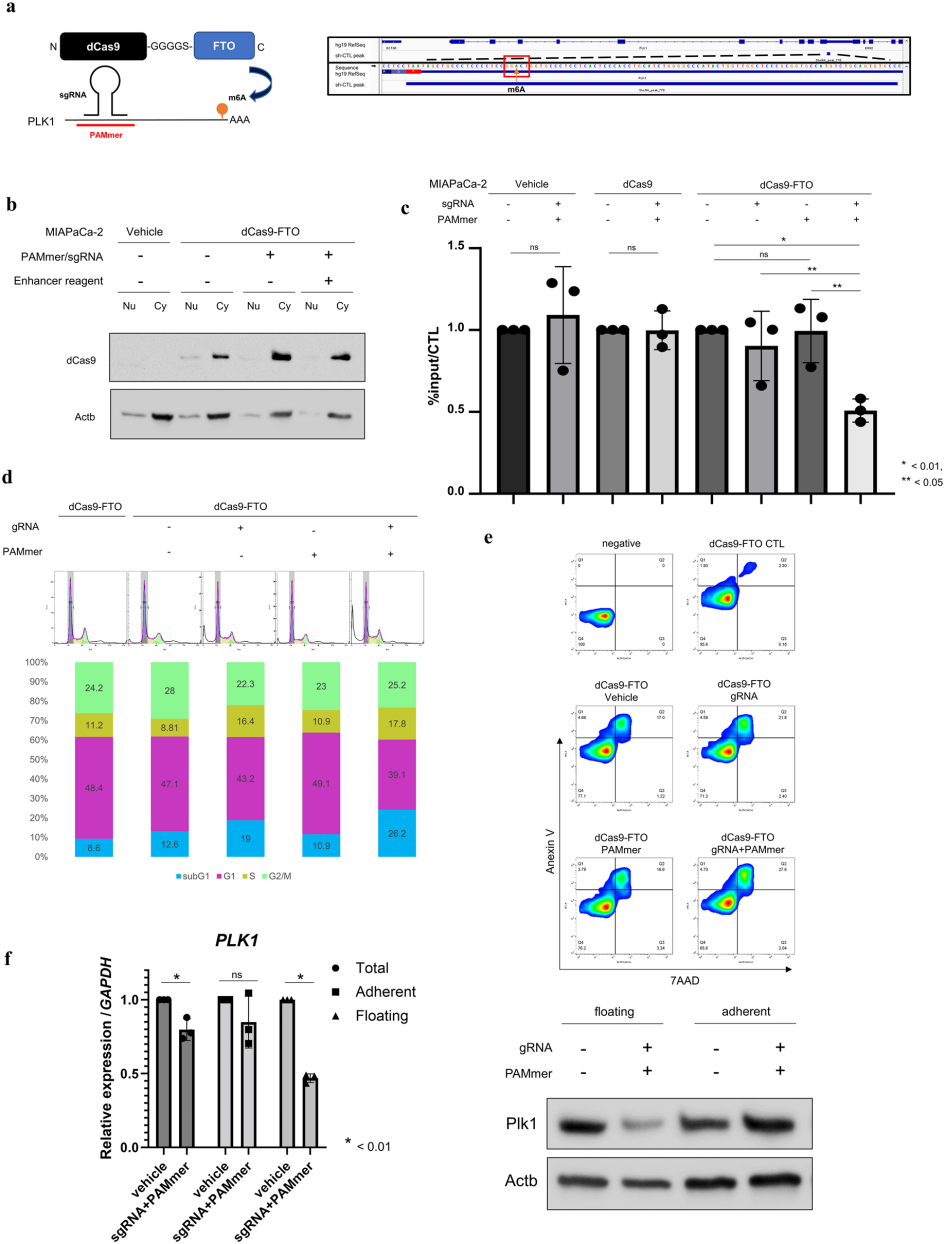
Site-specific RNA N6-methyladenosine demethylation by dCas9-FTO. (**a**) The dCas9–FTO fusion protein is guided to the respective target RNA via sgRNA and PAMmer to recruit it close to the m6A site for targeted demethylation. The figure on the right shows the methylation sites being targeted. (**b**) Confirmation that dCas9–FTO acts in the cytoplasm. When sgRNA and PAMmer were transfected, dCas9–FTO, which was present in the nucleus, was also transferred to the cytoplasm. This indicates that dCas9 acts on the target mRNA in the cytoplasm. Uncropped images are shown in Supplementary Fig. 7. (**c**) MeRIP–qPCR analysis shows that in the dCas9–FTO system, co-transfection of sgRNA and PAMmer reduces methylation of the PLK1 3′UTR. The degree of methylation is indicated by the %input in the vehicle transfection of each cell line (vehicle, dCas9 only, and dCas9–FTO, respectively). Values are the mean ± standard deviation of 3 and the *p* values in *p < 0.01 and **p < 0.05 were determined by two-tailed paired-samples t-tests. (**d**) Cell cycle changes induced by PLK1 3′UTR demethylation. Co-transfection of sgRNA and PAMmer increased not only the S–G2–M phase but also the sub-G1 peak. (**e**) Annexin V staining assay indicates that PLK1 3′UTR demethylation increased Annexin V- and 7-AAD-positive populations, which are at the terminal stage of cell death. (**f**) Altered expression of PLK1 by PLK1 3′UTR demethylation. No significant variation in PLK1 expression in adherent cells was observed 48 h after the transfection; however, PLK1 expression was significantly decreased in floating cells (left: mRNA, right: protein). Uncropped images are shown in Supplementary Fig. 7.

We performed site-specific RNA N6-methyladenosine (m6A) editing to evaluate the effects of PLK1 3′UTR methylation. We constructed a plasmid as previously reported^9^ and established nuclease-deactivated Cas9 (dCas9)–alpha-ketoglutarate-dependent dioxygenase (FTO) stably expressing MIAPaCa-2. In addition to the cell line, we established two cell lines: one overexpresses dCas9 only and the other overexpresses neomycin resistance gene only (vehicle) as controls (Fig. 6a; Supplementary Fig. 5b,c). The targeted m6A site was located at chr16: 23701404 (hg19), and we designed single-guide RNA (sgRNA) and protospacer adjacent motif (PAM)-presenting oligonucleotide (PAMmer) targeting m6A (Supplementary Data 1). As a proof of concept, we examined the subcellular localization of dCas9–FTO using Western blotting in the nucleus and cytoplasm separately after transfection of sgRNA and PAMmer (Fig. 6b). The results showed that compared with the control (vehicle), dCas9–FTO, which was present in the nucleus, was transferred to the cytoplasm in the sgRNA/PAMmer-transfected group with or without an enhancer regent. This indicates that dCas9–FTO acts on its target RNA in the cytoplasm in the presence of sgRNA and PAMmer^40^. Then, MeRIP-quantitative polymerase chain reaction (qPCR) was performed to quantify the methylation of the PLK1 3′UTR (Fig. 6c). After optimizing the RNA fragmentation size and primers (Supplementary Fig. 5d), sgRNA only, PAMmer only, and sgRNA/PAMmer transfections were performed using vehicle and dCas9 overexpression cell lines as controls, and the rate of methylation change was calculated for each control (lipofection reagent only). The results showed no significant change in methylation between the two controls and either sgRNA or PAMmer alone (although sgRNA alone tended to slightly decrease methylation); however, a significant decrease in methylation in the cell line transfected with sgRNA and PAMmer was observed. The effect of demethylation of the PLK1 3′UTR increased the S/G2/M phase and the number of cells in the subG1 phase (Fig. 6d), and evaluation using trypan blue showed a marked decrease in total cell count and viability (Supplementary Fig. 6a). It suggested that cell death, including apoptosis, was increased, which was evaluated by annexin V and 7-aminoactinomycin (AAD) assay, and it was found that the demethylation of the PLK1 3′UTR increased the number of annexin V- and 7-AAD-positive cells, suggesting an increase in late apoptosis (Fig. 6e). Confirming the variation in PLK1 expression due to the decrease in methylation of the PLK1 3′UTR, there was no significant variation in PLK1 expression in adherent cells 48 h after the transfection; however, PLK1 expression was significantly decreased in floating cells (Fig. 6f).

### The demethylation of the PLK1 3′UTR causes replication stress via dissociation of IGF2BP2, leading to mitotic catastrophe and enhanced radiosensitivity.

**Figure 7:**
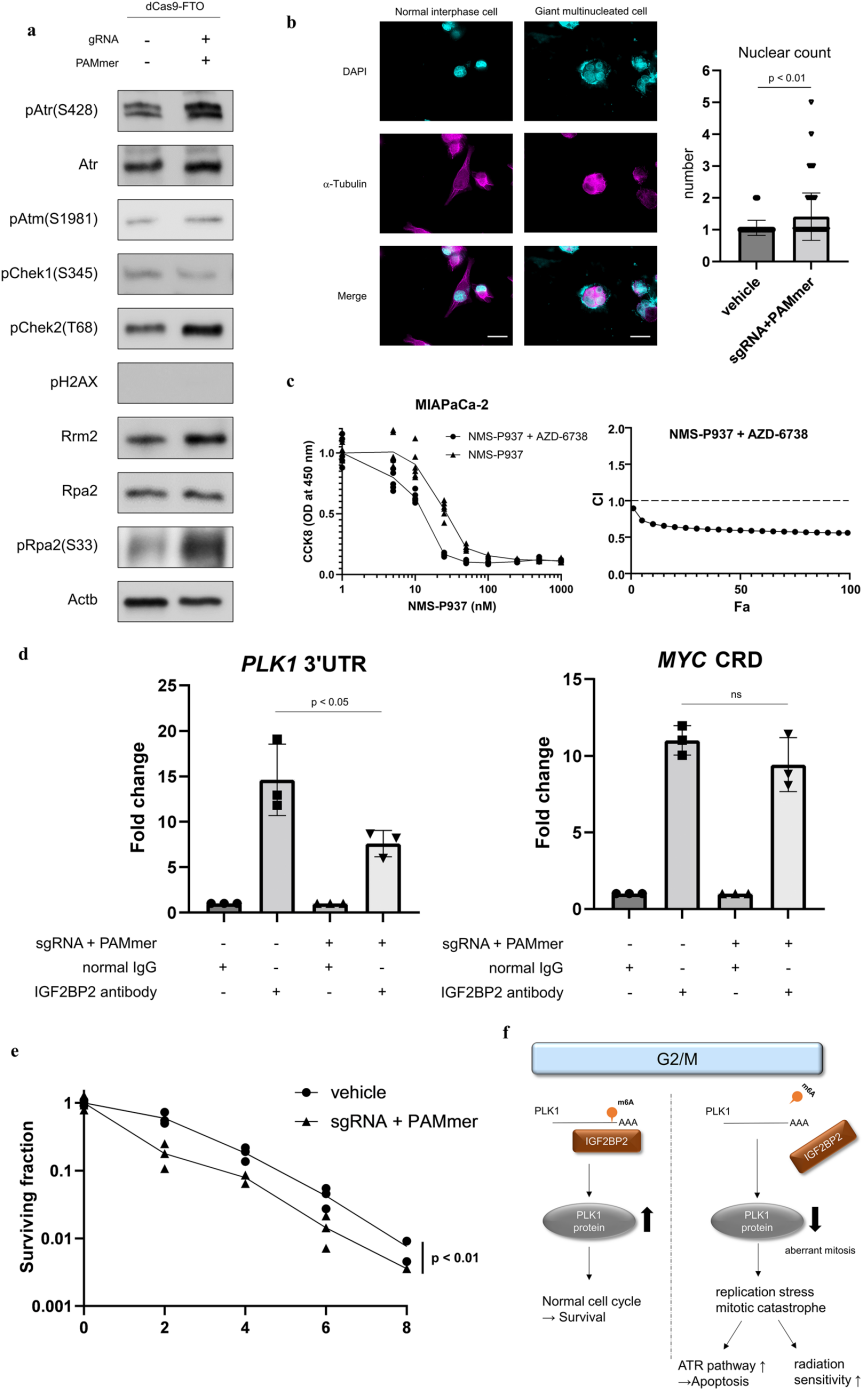
Demethylation of the PLK1 3′UTR causes replication stress via dissociation of IGF2BP2, leading to mitotic catastrophe and enhanced radiosensitivity. (**a**) Western blotting to elucidate the mechanism of cell death by PLK1 3′UTR demethylation. Uncropped images are shown in Supplementary Fig. 7. (**b**) Immunocytochemistry to assess mitotic catastrophe. The left shows microscopic images (DAPI: cyan; α-Tubulin: magenta) of cells treated by vehicle or sgRNA/PAMmer, and the latter shows a typical mitotic catastrophe. In the right panel, 200 interphase cells were randomly extracted, and the number of nuclei in multinucleated cells is compared between vehicles and sgRNA/PAMmer transfection. The statistical analysis was performed by Student’s t-test. The scale represents 20 µm. (**c**) Synergistic effect of NMS-P937, a PLK1 inhibitor, and AZD-6738, an ATR inhibitor. On the left, the sigmoid curve of cytotoxicity is evaluated using CCK8. On the right side, the synergistic effect is evaluated using the combination index. This (C.I. < 1.0) indicates the synergistic effect of NMS-P937 and AZD-6738 in MIAPaCa-2. (**d**) RIP–qPCR with IGF2BP2 showed that PLK1 3′UTR demethylation reduced IGF2BP2 binding. In MYC, where IGF2BP2 binds, IGF2BP2 binding was not altered by sgRNA/PAMmer transfection. (**e**) A clonogenic assay to evaluate changes in radiosensitivity due to PLK1 3′UTR demethylation. Values are the mean ± SD of 3 and the *p* value in *p* < 0.01 was determined by ANOVA based tests using R package “CFAssay”. (**f**) The m6A–IGF2BP2–PLK1 pathway is important for maintaining the cell cycle in pancreatic adenocarcinoma.

To elucidate the mechanism of cell death caused by demethylation of the PLK1 3′UTR, we analyzed the signaling pathways related to the DNA damage response using Western blotting and found that the transfection of sgRNA and PAMmer increased ribonucleotide reductase regulatory subunit M2 and phosphorylated replication protein A2 (RPA2), suggesting that replication stress is caused by PLK1 3′UTR demethylation (Fig. 7a). Furthermore, replication stress induced the activation of ATR, which in turn causes CHEK2 phosphorylation^58,59^.

Next, we performed immunocytochemistry to analyze the mitotic catastrophe assumed to be the result of mitotic defects by decreased expression of PLK1 due to the demethylation of 3′UTR (Fig. 7b). The left side of Fig. 7b shows the typical morphological features of mitotic catastrophe, a giant multinucleated cell following mitotic catastrophe, induced by the demethylation of PLK1 3′UTR via sgRNA/PAMmer transfection. To compare the frequency of mitotic catastrophe with and without the demethylation of the PLK1 3′UTR, 200 interphase cells were extracted in each condition (vehicle or sgRNA/PAMmer), and the number of nuclei was measured automatically. The number of nuclei per cell was significantly higher in sgRNA/PAMmer-transfected cells than that in vehicles. This indicates that mitotic catastrophe is involved in cell death caused by PLK1 3′UTR demethylation.

These results suggest that the demethylation of the PLK1 3′UTR causes mitotic catastrophe by downregulating PLK1 expression in the G2/M phase, in which PLK1 upregulation is required, resulting in aberrant mitosis and replication stress, followed by the activation of the ATR–CHEK2 axis^60–62^. As the inhibition of WEE1 G2 checkpoint kinase (WEE1), a downstream PLK1 protein that is important for mitosis progression, and ATR is effective in breast cancer and other cell lines^63^, the combination of AZD-6738, an ATR inhibitor, and NMS-P937, a PLK1 inhibitor, was effective and synergistic in MIAPaCa-2 (Fig. 7c). This is due to the inhibition of ATR, which is important for recovery from replication stress caused by PLK1 inhibition^58,62^. Next, to search for the reader protein for the methylated PLK1 3′UTR, we analyzed the following public databases: ENCORI and POSTAR2^64,65^. Among the proteins that bind to the same site as the methylation, insulin like growth factor 2 mRNA binding protein 2 (Igf2bp2), Lin-28 homolog B (Lin28b), cleavage stimulation factor subunit 2 Tau variant (Cstf2t), embryonic lethal, abnormal vision, Drosophila, homolog-like 1 (Elavl1), and Fragile X mental retardation protein 1 (Fmr1) were extracted on the condition that they are included in both databases and are highly reliable. Among those candidates, IGF2BP2 was the only one associated with poor prognosis in patients with pancreatic adenocarcinoma at high expression levels (from TCGA, data not shown), and the highest correlation was observed between the pathological grade and PLK1 expression level, so IGF2BP2 was considered the most likely candidate of methylation leader proteins of the PLK1 3′UTR (Supplementary Fig. 6b). Other reports have shown that IGF2BP2 is an RNA-binding protein that upregulates SRY-box transcription factor 2 (SOX2) expression methylated by METTL3 in colon carcinoma^31^. Furthermore, the public data (GSM2409783), which performed RIP using IGF2BP2 at HEK293T cells, confirmed that IGF2BP2 was indeed bound to the PLK1 3′UTR site (Supplementary Fig. 6c). To confirm this, we performed RNA immunoprecipitation using IGF2BP2. The demethylation of the PLK1 3′UTR did not alter the region, v-myc Avian myelocytomatosis viral oncogene homolog (c-myc) mRNA coding region instability determinant (MYC CRD), as a methylated RNA-binding site for IGF2BP2; however, IGF2BP2 binding to the PLK1 3′UTR was reduced (Fig. 7d). This indicates that IGF2BP2 acts as a methylated RNA-binding protein to the PLK1 3′UTR. Finally, we performed a clonogenic assay to evaluate changes in radiosensitivity due to PLK1 3′UTR demethylation. The results showed that PLK1 3′UTR demethylation enhanced radiosensitivity, reflecting enhanced replication stress (Fig. 7e).

## Discussion

In our previous study, we reported that high expression of METTL3 in pancreatic cancer cell lines is associated with resistance to chemotherapy and radiation^7^, and in this study, we elucidated the mechanism in detail. We found that PLK1 is the most important gene whose methylation and expression are directly regulated by METTL3 and that METTL3 expression fluctuates in correlation with PLK1 in a cell cycle-dependent manner. Furthermore, the methylation of the PLK1 3′UTR stabilizes the expression of PLK1 by binding IGF2BP2 and is essential for cell cycle homeostasis, and its demethylation leads to replication stress-mediated cell death and increased radiosensitivity by activating the ATR pathway (Fig. 7f).

The relationship between m6A, the most common methylation modifier of RNA, and malignant tumors has been reported recently; however, whether it acts as an oncogene or a tumor suppressor gene has been reported differently because the target genes of methylation vary among malignant tumors^66^. For example, in glioblastoma, ADAM19 expression is upregulated in association with the downregulation of METTL3 expression, which promotes self-renewal of cancer stem cells^67^, whereas, in lung adenocarcinoma, RNA methylation stabilizes epidermal growth factor receptor expression and is involved in tumor growth and invasion^68^. The role of METTL3 in pancreatic cancer was first reported in our previous study, and although METTL3 is higher in pancreatic adenocarcinoma than in healthy pancreatic tissue and patients with high METTL3 levels have a poor clinical prognosis^69^, the detailed mechanism by which METTL3 methylates and regulates the expression of genes has not yet been elucidated in pancreatic adenocarcinoma. This study revealed that METTL3 regulated cell cycle, especially mitosis, through the methylation of those genes in pancreatic cancer cells, and PLK1 was identified as a gene playing a central role in this process. Recently, a comprehensive study on pancreatic adenocarcinoma using a database of genes involved in RNA methylation has been conducted, and PLK1 has been extracted as one of the genes involved in RNA methylation^70^. However, the detailed mechanism of how it is involved in promoting pancreatic adenocarcinoma has not been investigated so far.

PLK1 has long been considered an oncogene because of its role in cell proliferation; however, recently, it acts as a tumor suppressor in some carcinomas^71^. For example, it acts as an oncogene in estrogen receptor-positive breast cancer^72^, while it has also been reported to act as a tumor suppressor under special circumstances as in adenomatous polyposis coli-deficient colon cancer^73^. PLK1 is upregulated in pancreatic adenocarcinoma and has long been considered a therapeutic target^74^. In TCGA data analysis in this study, PLK1 works predominantly as an oncogene in pancreatic adenocarcinoma, and its elevated expression is associated with worse prognosis. Functional analysis of PLK1 in pancreatic cancer cells showed that NMS-P937, a PLK1 inhibitor, could efficiently induce G2/M arrest, and along with radiation, it had a synergistic effect by increasing DNA double strand breaks. Furthermore, analysis of a mouse model of pancreatic carcinogenesis that overexpresses METTL3 indicates that the upregulated effect of cell cycle by METTL3 is tumor cell-specific and that the antitumor effect of PLK1 inhibition is stronger in tumor cells compared with normal pancreatic duct cell. As a point that require attention in interpretation, we noted that the pancreatic tumors in mice indicated acinar cell carcinoma-like features, whereas human pancreatic cancer showed histology of ductal adenocarcinoma, implying that we studied the effect of METTL3 overexpression in vivo in a mouse model that was close to the spontaneous occurrence of tumors, and suggesting that PLK1 is a useful therapeutic target for pancreatic adenocarcinoma, which is refractory to treatment.

Several studies have shown that METTL3 regulated the cell cycle through various pathways. METTL3 regulates the cell cycle during adipocyte differentiation by regulating cyclin A2^75^, and METTL3 KD significantly reduces G0/G1 arrest by lowering p21 protein expression in renal cell carcinoma^76^. Moreover, in dental pulp stem cells, METTL3 KD affects the cell cycle and causes fluctuations in the expression of PLK1^77^. We showed that METTL3 could be involved in cell cycle-dependent expression fluctuations and upregulation of PLK1 expression via PLK1 3′UTR methylation.

In this study, we developed a site-specific RNA demethylation system by applying the CRISPR-Cas9 system^9^ and examined the various changes in pancreatic cancer cells by demethylating the PLK1 3′UTR site with FTO, increasing cell death with an increase in the S–G2–M phase. This was attributed to replication stress caused by aberrant mitosis due to decreased expression of PLK1 at the G2/M phase^62,78^, leading to mitotic catastrophe. Furthermore, the state of aberrant mitosis could enhance radiosensitivity and contribute to improving radioresistance in pancreatic adenocarcinoma. Aurora kinase is the main cascade for PLK1 activation^79^; however, the involvement of the HER2–SHCBP1 pathway has recently been reported in gastric cancer^80^, suggesting that there are variations depending on the cancer type. In this study, we suggest a possibility of the mechanism for stabilizing PLK1 expression by the METTL3–IGF2BP2 pathway in pancreatic adenocarcinoma, which may be a new therapeutic target.

Small molecule compounds that inhibit METTL3 have been developed and shown to be useful in hematological malignancies^81^. These compounds were not useful for pancreatic cancer cell lines (data not shown) because they reduce overall m6A methylation and have various target genes. If site-specific PLK1 demethylation can be performed in the future, applying replication stress only to mitotic cancer cells may be possible, and in combination with radiotherapy, achieving tumor-specific radiosensitization with less toxicity to healthy tissues may be possible. Furthermore, WEE1, the downstream signal of PLK1, and ATR inhibitors may be effective therapies in breast cancer cell lines^63^, and in this study, we found the possibility of combining PLK1 and ATR inhibition by the common mechanism. This may be an effective treatment for patients with multiple metastases or other diseases that are difficult to treat using radiotherapy.

In conclusion, METTL3 regulates the cell cycle in pancreatic adenocarcinoma cells through the methylation of the PLK1 3′UTR, and its disruption of homeostasis increases cell death via replication stress and may be a new target for radiosensitization.

## Supplementary Information

Supplementary Figures.

Supplementary Information 1.

Supplementary Information 2.

Supplementary Information 3.

Supplementary Information 4.

Supplementary Information 5.

## Data Availability

The authors declare that all data supporting the findings of this study are available within the article, the supplementary data, and the data repository or from the corresponding author upon reasonable request. The RNA sequencing data are deposited in the Gene Expression Omnibus of the National Center for Biotechnology Information (accession no. GSE190078, GSE202004, GSE202005, and GSE202113).
