# Supplementary material for: N(6)-methyladenosine methylation-regulated polo-like kinase 1 cell cycle homeostasis as a potential target of radiotherapy in pancreatic adenocarcinoma
Source: Sci Rep. 2022 Jun 30;12:11074. doi: 10.1038/s41598-022-15196-5 (PMC9246847; doi:10.1038/s41598-022-15196-5)
Supplement: Supplementary file 1 — Supplementary Figures. [file 41598_2022_15196_MOESM1_ESM.docx]

**Supplementary Figure Legends**

**N(6)-methyladenosine methylation-regulated polo-like kinase 1 cell cycle homeostasis as a potential target of radiotherapy in pancreatic adenocarcinoma**

Shotaro Tatekawa, Keisuke Tamari, Ryota Chijimatsu, Masamitsu Konno, Daisuke Motooka, Suguru Mitsufuji, Hirofumi Akita, Shogo Kobayashi, Tetsuya Sato, Yoshiki Murakumo, Yuichiro Doki, Hidetoshi Eguchi, Hideshi Ishii, Kazuhiko Ogawa

**
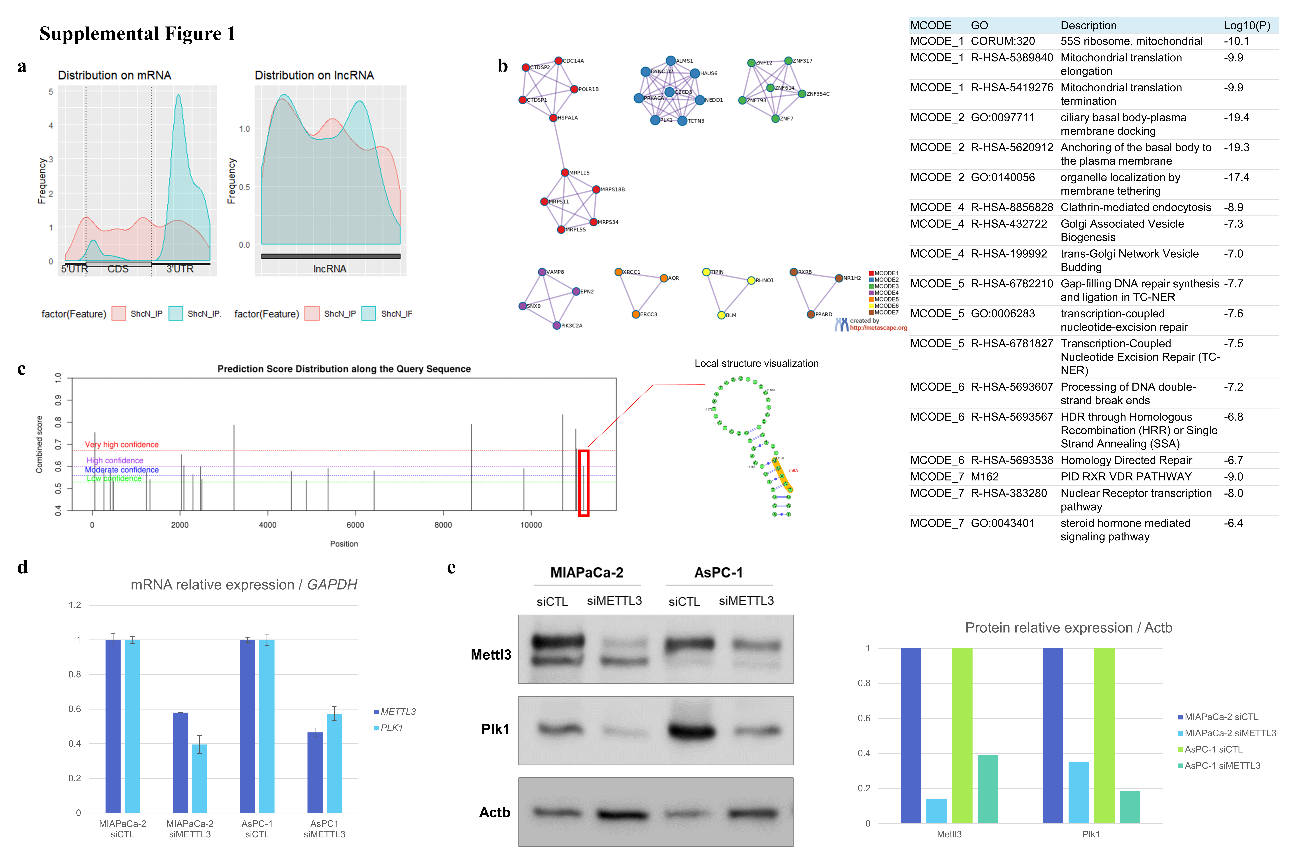
**

**Supplementary Fig. 1**

(a) Guitar plot of MeRIP-seq. The pink color shows the distribution of the entire peak, while cyan shows the distribution of significant peaks (fold change of more than 2 and q value of less than 0.01) at mRNA and lincRNA, respectively.

(b) Individual modules in MCODE in PPI analysis.

(c) The predicted score based on the nucleotide sequence of the methylation site. The red square indicates the methylation site of the PLK1 3'UTR (left), and the right indicates the local structure.

(d), (e) Expression changes of PLK1 in MIAPaCa-2 and AsPC-1 by METTL3-KD. Protein concentrations are also shown as densities quantified using ImageJ (right side of e).


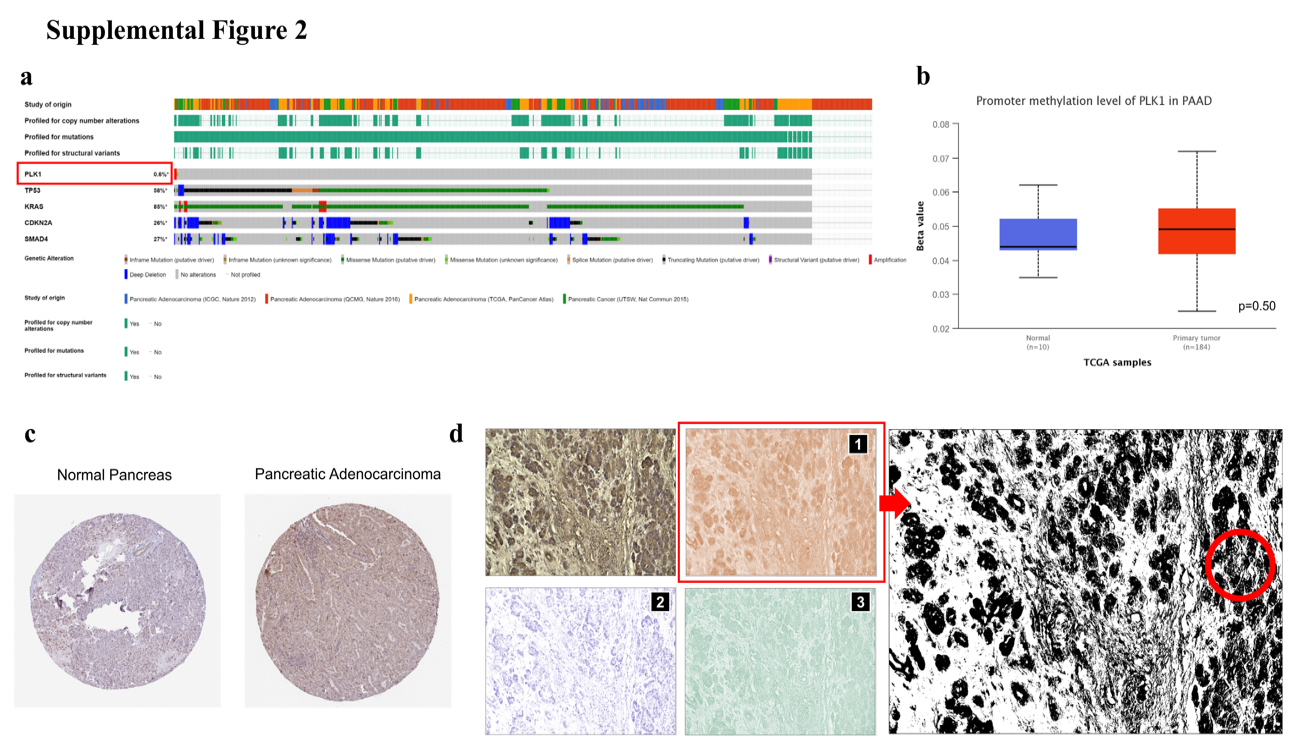


**Supplementary Fig. 2**

(a) Mutation lists of pancreatic adenocarcinoma in four studies (i.e., ICGC; Nature 2012, QCMG; Nature 2016, TCGA; PanCancer Atlas, and UTSW; Nat Commun 2015). Compared with TP53, KRAS, CDKN2A, and SMAD4, which are typical mutated genes in pancreatic adenocarcinoma, the probability of PLK1 mutation is extremely low (0.5%).

(b) Promotor methylation level of PLK1 in pancreatic adenocarcinoma from TCGA samples. This indicates no difference in the degree of methylation of the PLK1 promoter between healthy pancreatic tissue and pancreatic adenocarcinoma.

(c) PLK1 protein expression level in pancreatic adenocarcinoma and healthy pancreatic tissue from the Human Protein Atlas.

(d) Quantitative comparative analysis of PLK1 and METTL3 expression using Color Deconvolution, a plugin of ImageJ. Color Deconvolution divides the original picture to three segments (1: DAB, 2: hematoxylin, and 3: others). The DAB segments were binarized at a certain threshold, and the mean of the cancer cell-only sites was calculated in a circle of a certain area. Three sites were measured per patient, and the mean was compared between each patient.


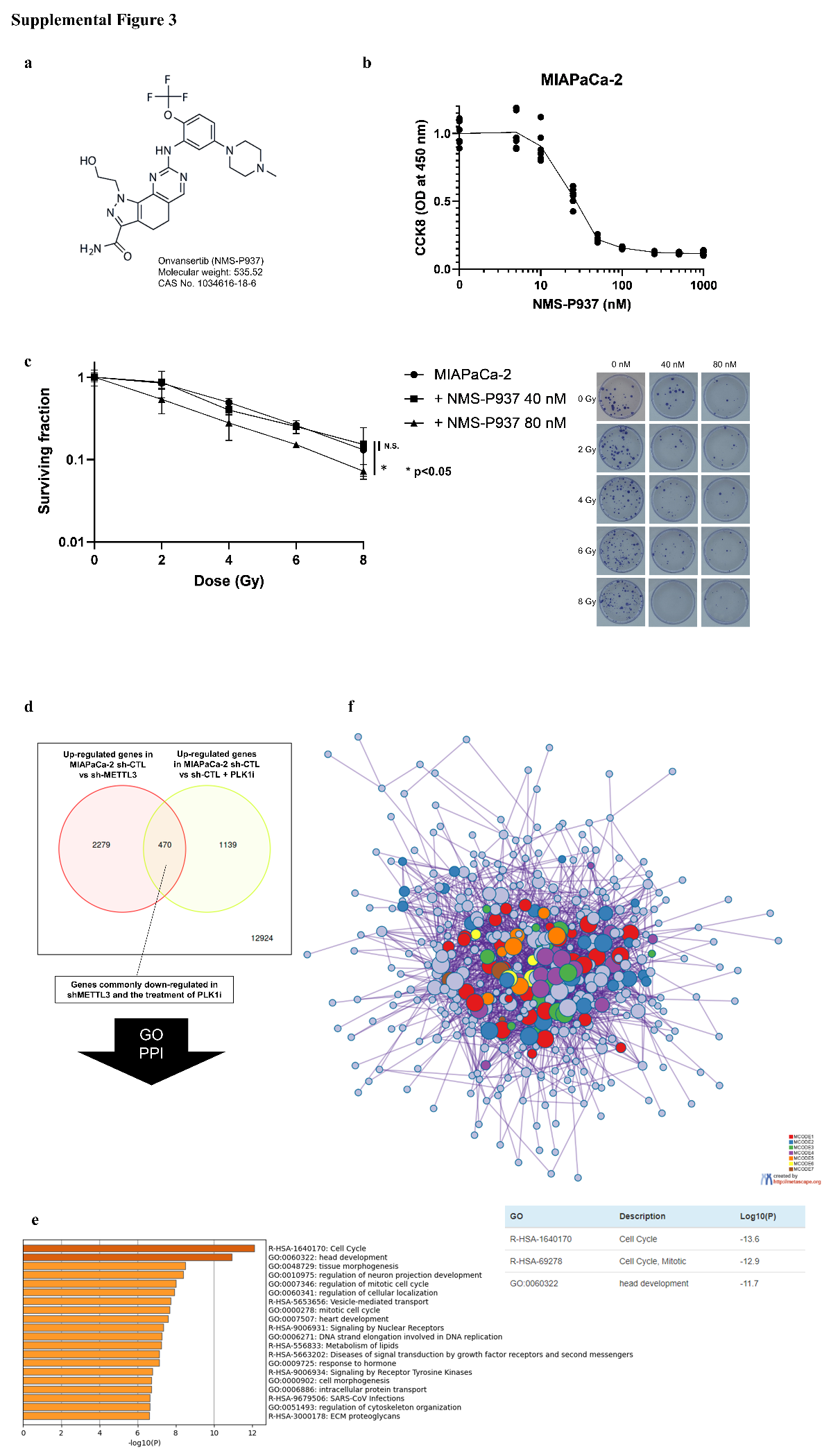


**Supplementary Fig. 3**

(a) The structural formula of NMS-P937.

(b) Cytotoxic effect of NMS-P937 on MIAPaCa2 evaluated using CCK8. NMS-P937 is effective for MIAPaCa2, and the IC50 is low (23.5 nM).

(c) A clonogenic assay to evaluate the synergistic effect of NMS-P937. When NMS-P937 is added at an effective concentration to induce G2/M arrest, it shows a synergistic effect with radiation; however, at lower concentrations, no synergistic effect is observed. This indicates that the synergistic effect is caused by G2/M arrest. Values are the mean ± SD of 3 and the p values in *p < 0.05 and **p <0.01 were determined by two-tailed paired-samples t-tests

(d) Benn diagram of down-regulated genes of MIAPaCa-2 sh-METTL3 and sh-CTL + PLKi compared with sh-CTL.

(e) GO and (f) PPI analysis show that there are many genes related with cell cycle in above genes.


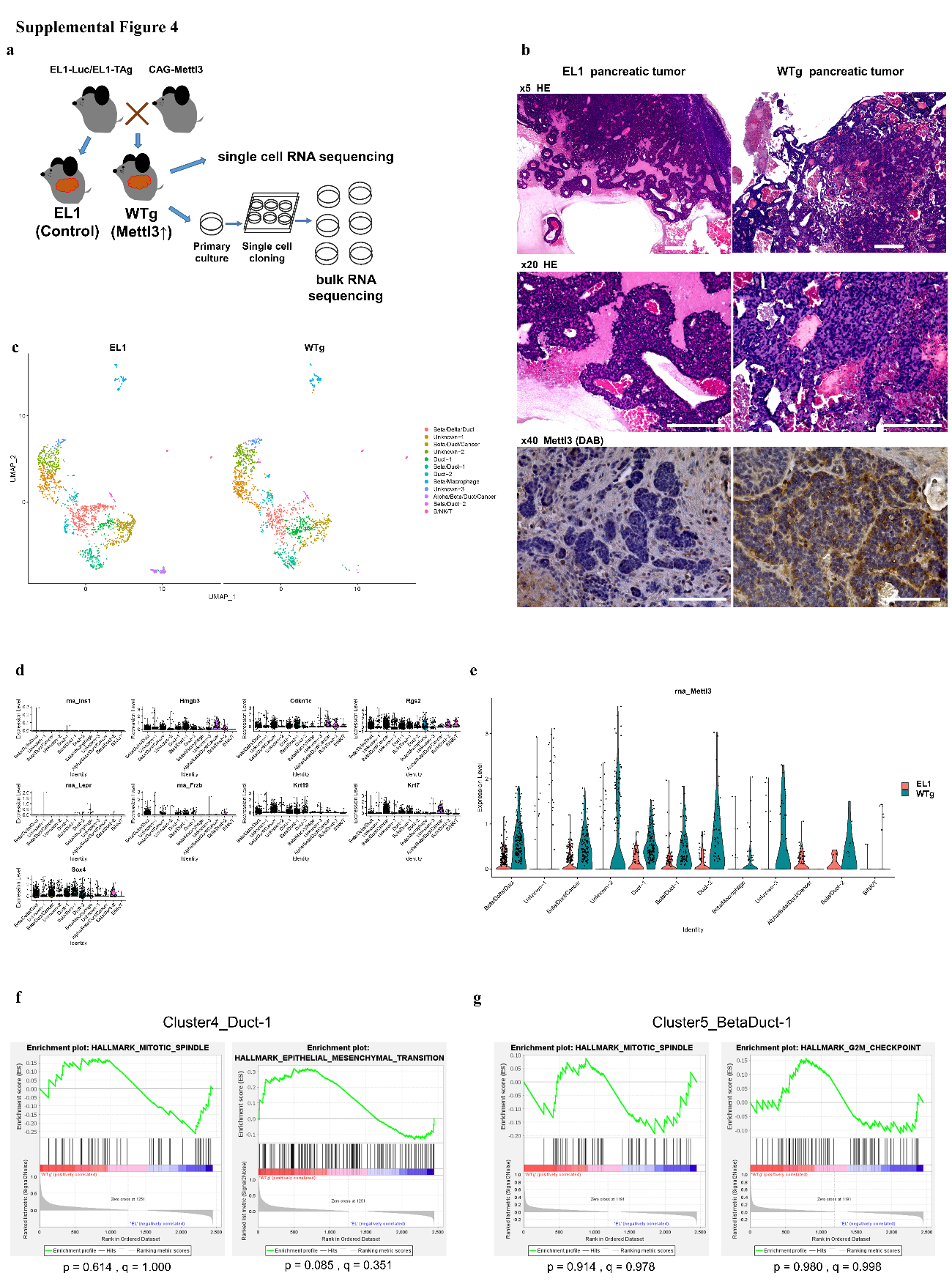


**Supplementary Fig. 4**

(a) The scheme of transgenic mice experiments.

(b) Histopathological images (Hematoxylin and Eosin staining and Mettl3 immunohistochemistry with DAB) of pancreatic tumors of EL1 (left side) and WTg (right side) mice. The scale represents 500, 200, and 100 μm, respectively, from the top.

(c) The data was summarized in the UMAP and color-coded according to cell type in EL1 (left) and WTg (right), respectively.

(d) The violin plot for each cluster in pancreas-related genes.

(e) Comparison of METTL3 expression between WTg and EL1 in each cluster.

(f, g) GSEA show the gene sets related with cell cycle were not enriched with WTg of the cluster of Duct-1 and Beta/Duct-1.

**
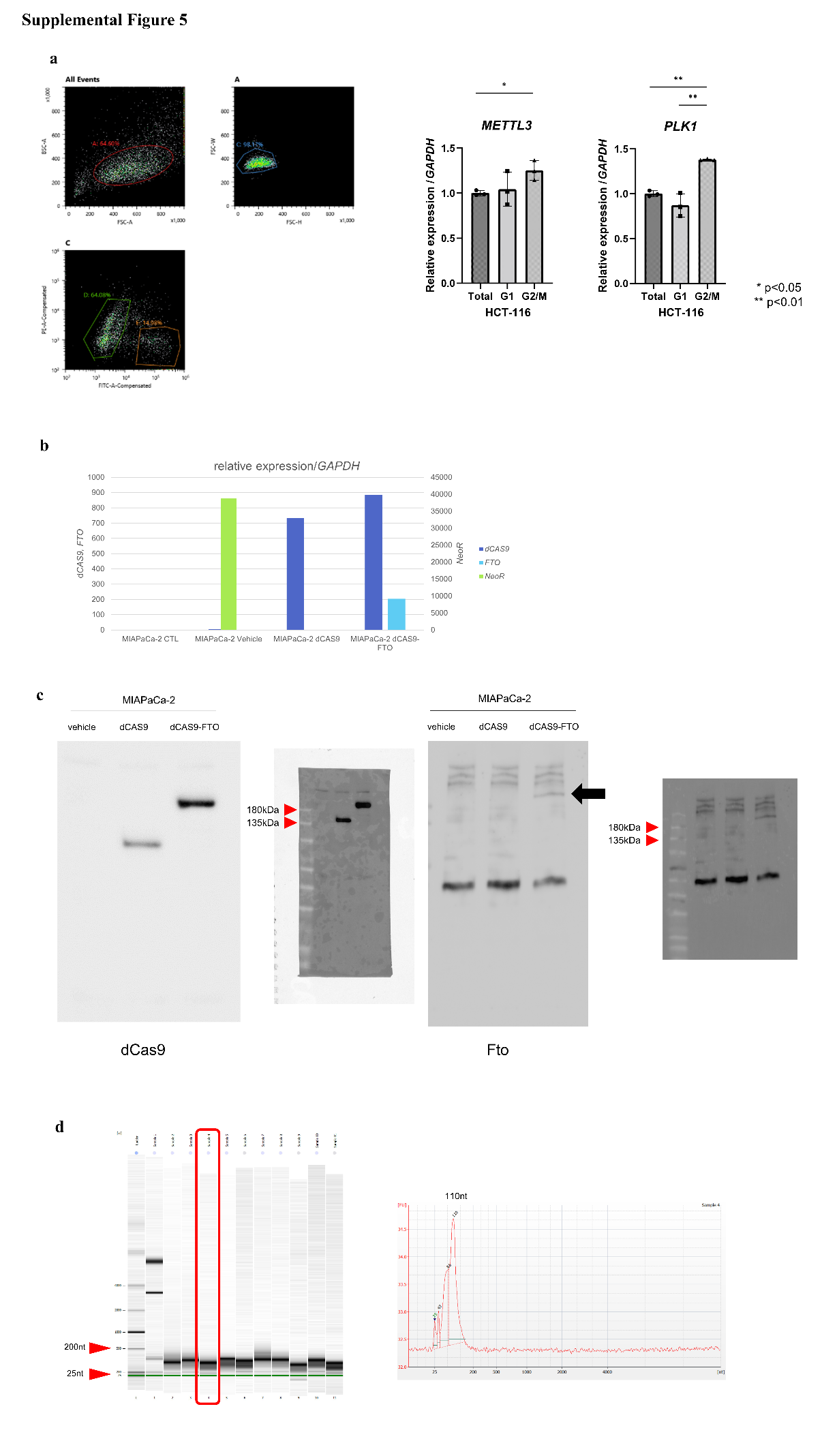
**

**Supplementary Fig. 5**

(a) Analysis of the changes in PLK1 and METTL3 expression in each cell cycle using FUCCI. PLK1 was upregulated in S–G2–M phase cells compared with that in pre-selection total cells and G1 phase cells, and METTL3 was upregulated in S–G2–M phase cells compared with that in total cells.

(b) Overexpression of dCas9 and FTO was confirmed using qPCR. The RNA sequence of overexpressed FTO was optimized (Supplementary Data 2), so endogenous FTO is undetected using PCR.

(c) Overexpression of dCas9 and FTO was confirmed by Western blotting. The protein sizes of dCas9 and FTO are 166 kDa and 58 kDa, respectively. The complex protein of dCas9 and FTO was observed at approximately 230 kDa (black arrow) with endogenous FTO whose size is 58 kDa.

(d) RNA fragmentation was examined under various conditions. The reaction time of ZnCl_2_ was changed step by step, and the reaction time of 90°C for 2 min was judged to be optimal because it resulted in a peak of approximately 110–120 nt.

**
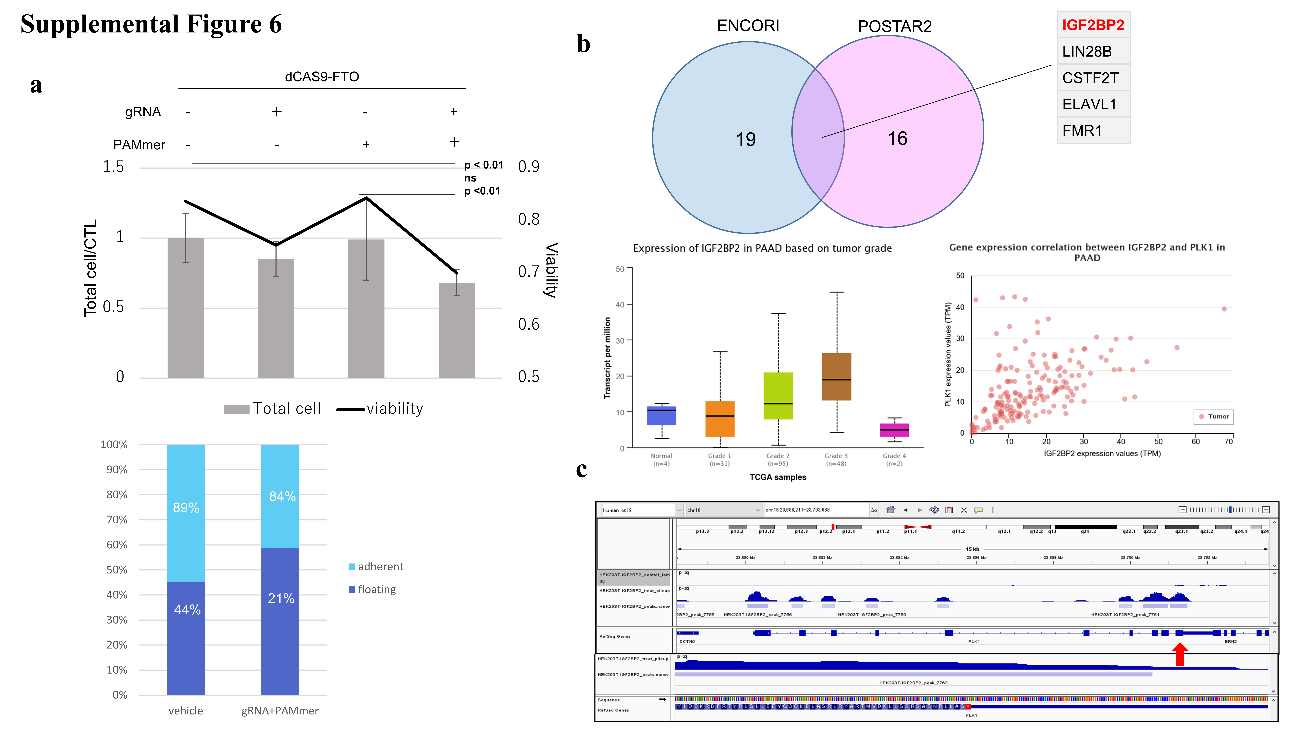
**

**Supplementary Fig. 6**

(a) Cell counting and viability assessment using trypan blue. The numbers in the bar graph represent the viability (lower). Values are the mean ± SD of 3.

(b) Search for RBPs that bind to the PLK1 3'UTR using a database. Using ENCORI and POSTAR2, Igf2bp2, Lin28b, Cstf2t, Elavl1, and Fmr1 were identified as RBPs with high confidence and predicted binding to the PLK1 3'UTR (upper). Among them, IGF2BP2 was the most associated with tissue malignancy and co-expression with PLK1 (lower).

(c) Visualization of the peak from data analysis performed using RIP-seq on IGF2BP2. We analyzed the public data (GSM2409783), which performed RIP with IGF2BP2 at HEK293T, and confirmed that IGF2BP2 was indeed bound to the PLK1 3'UTR site.


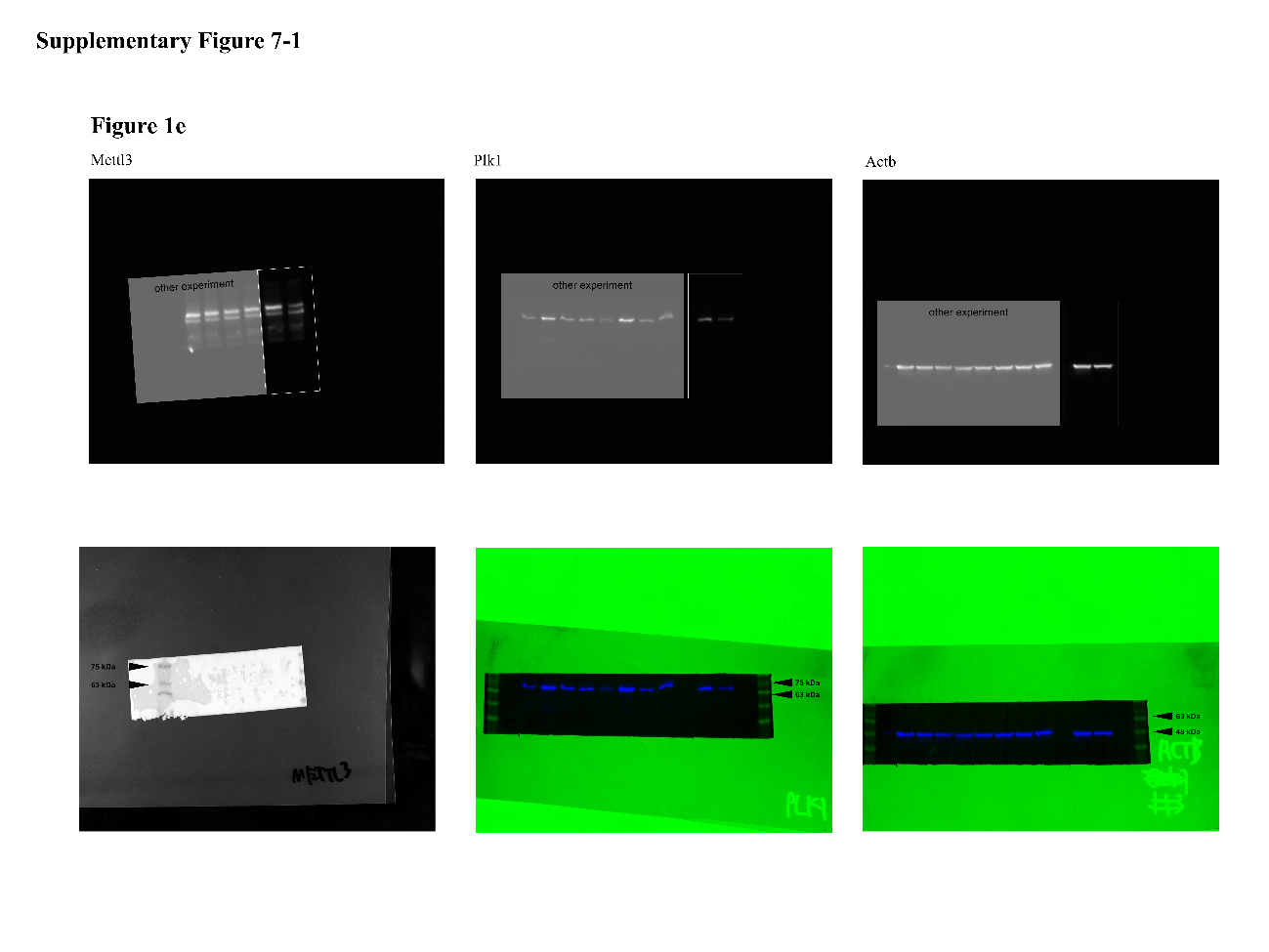


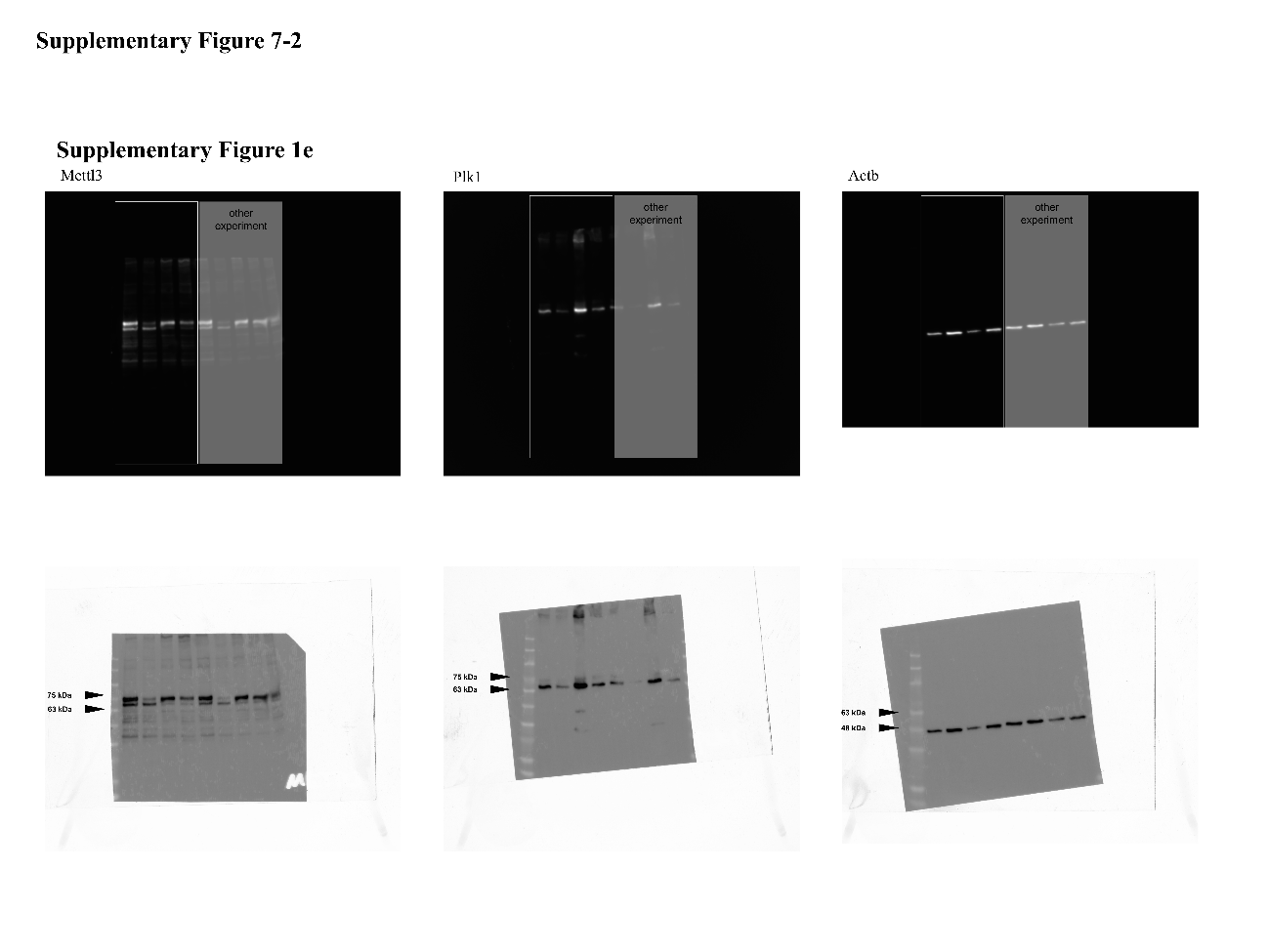


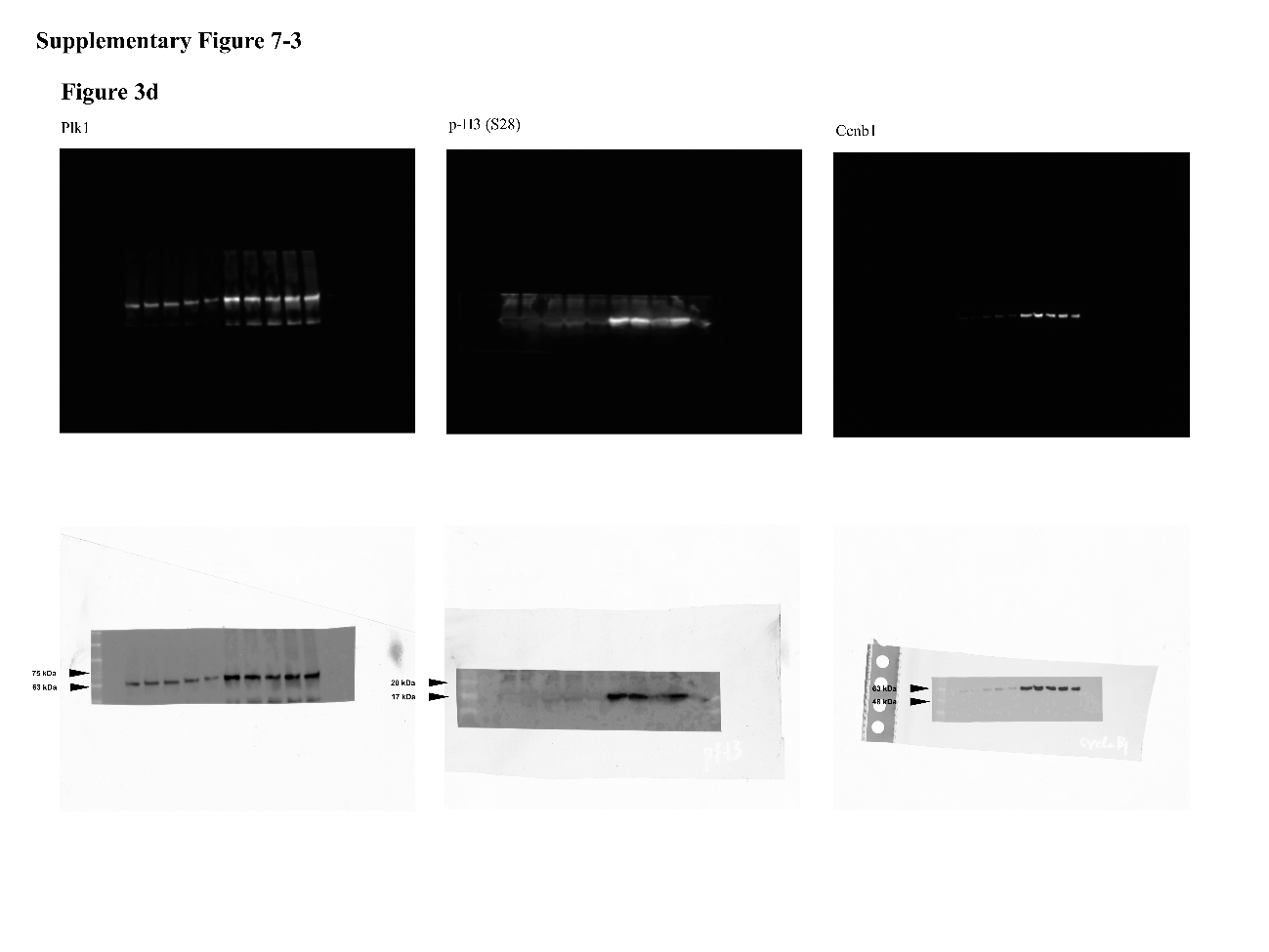


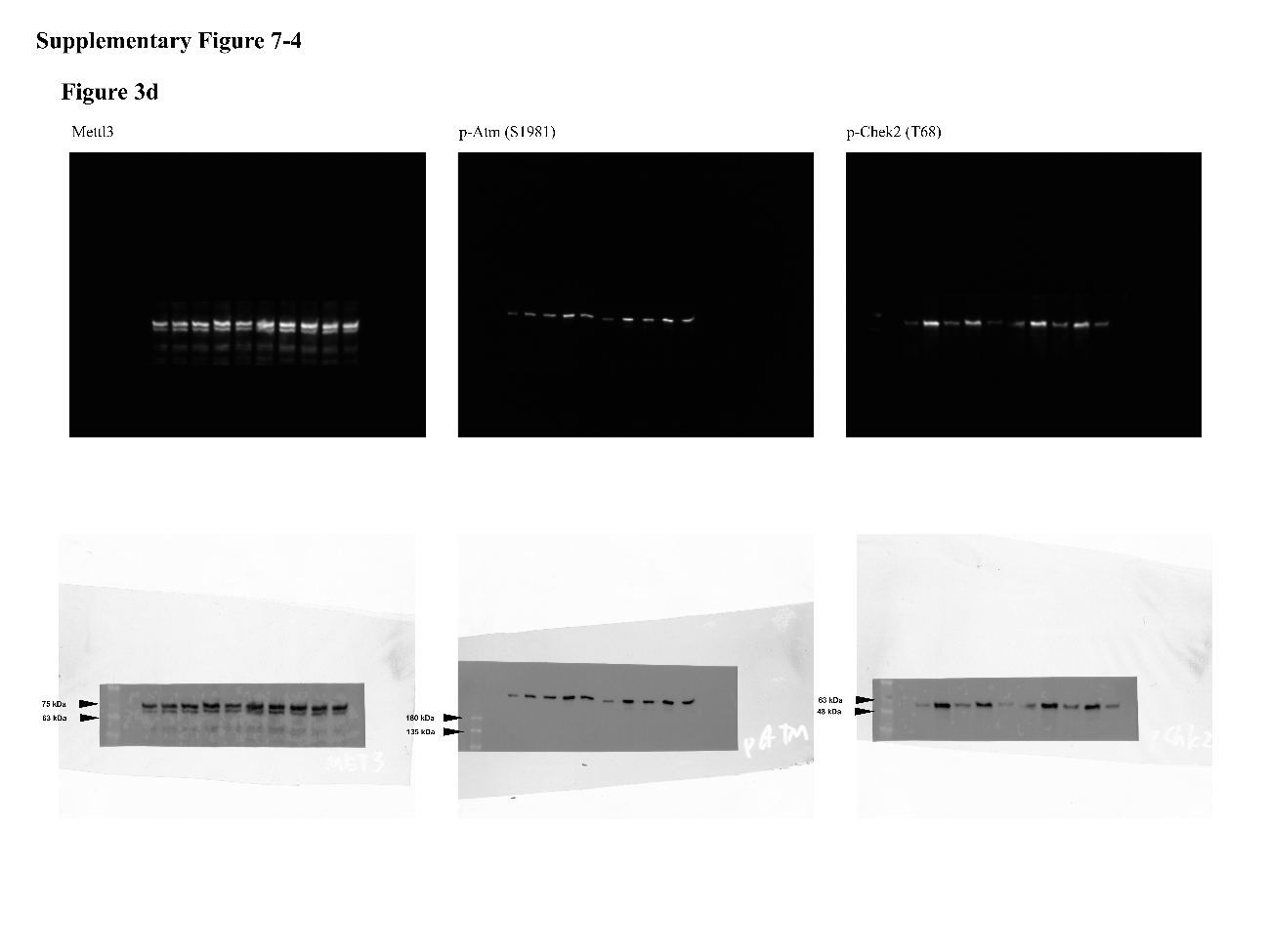


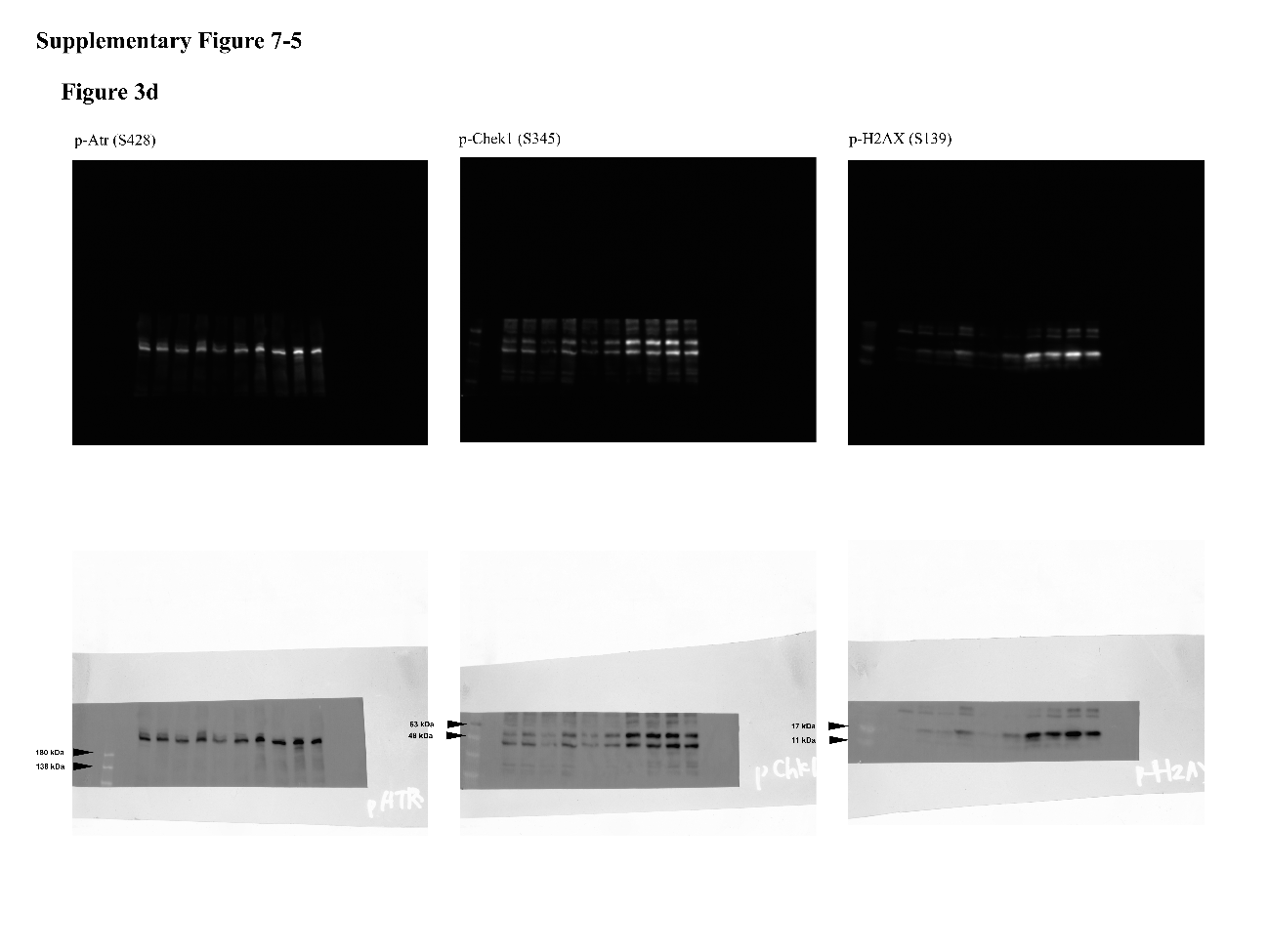


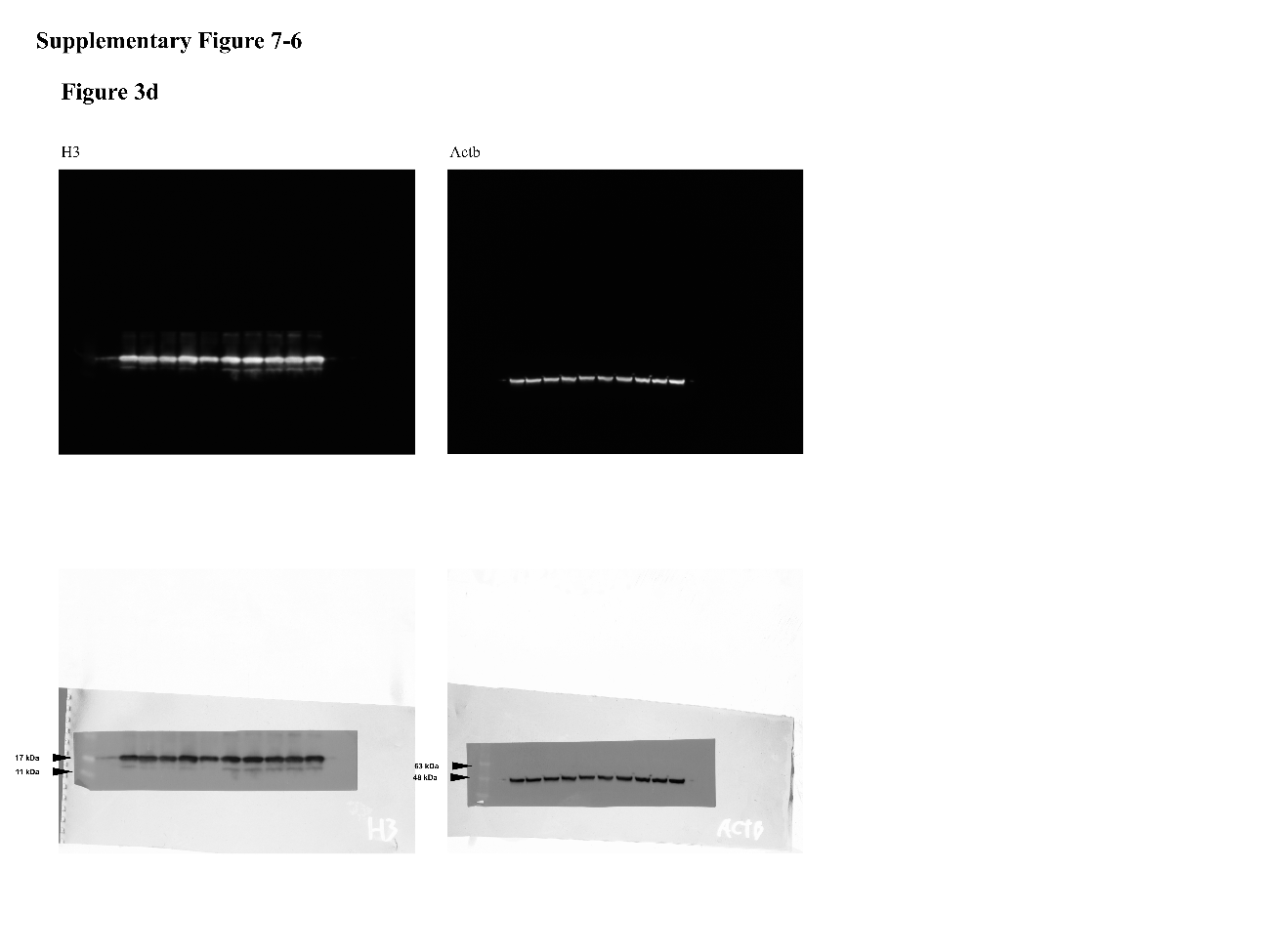


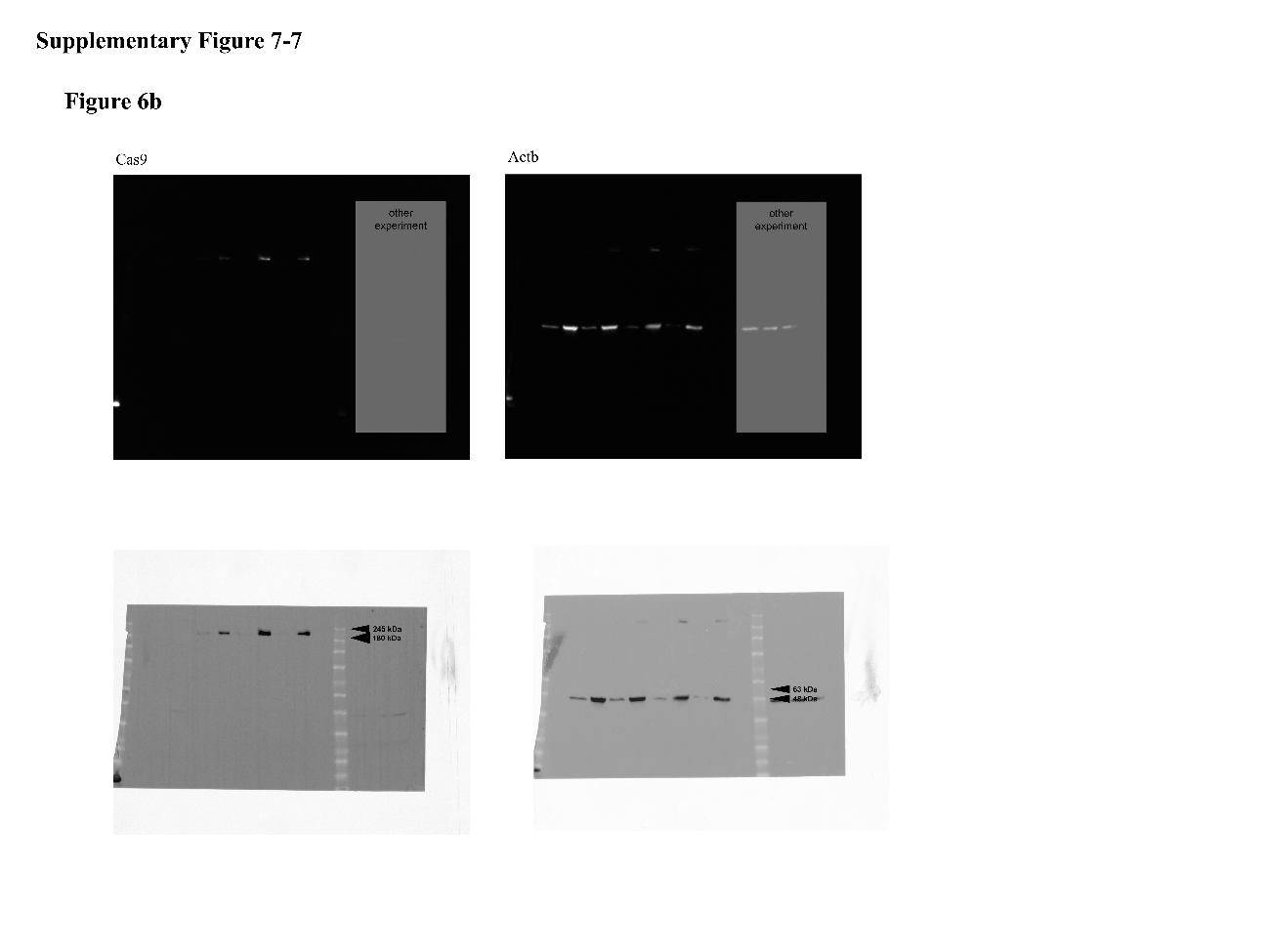


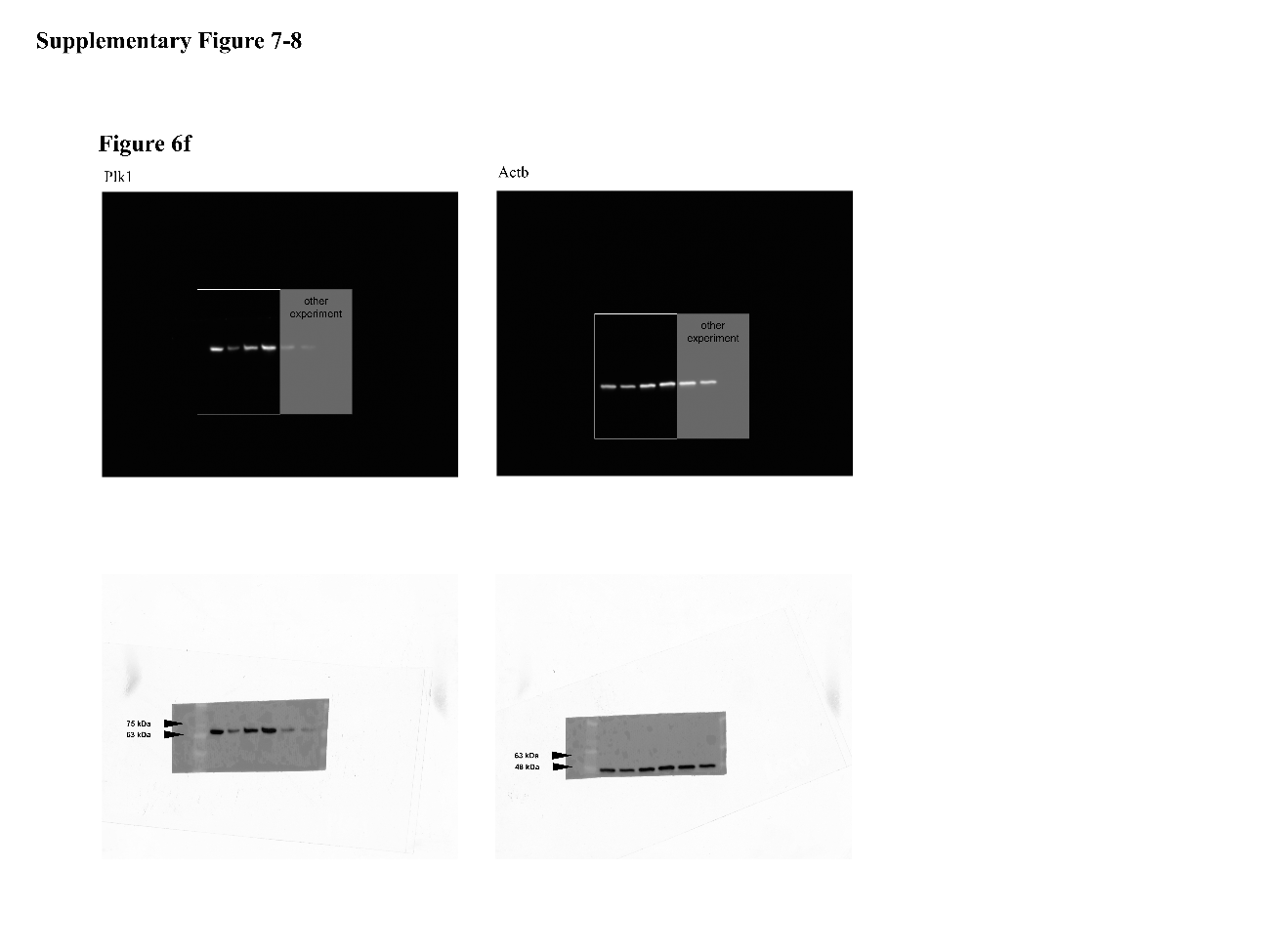


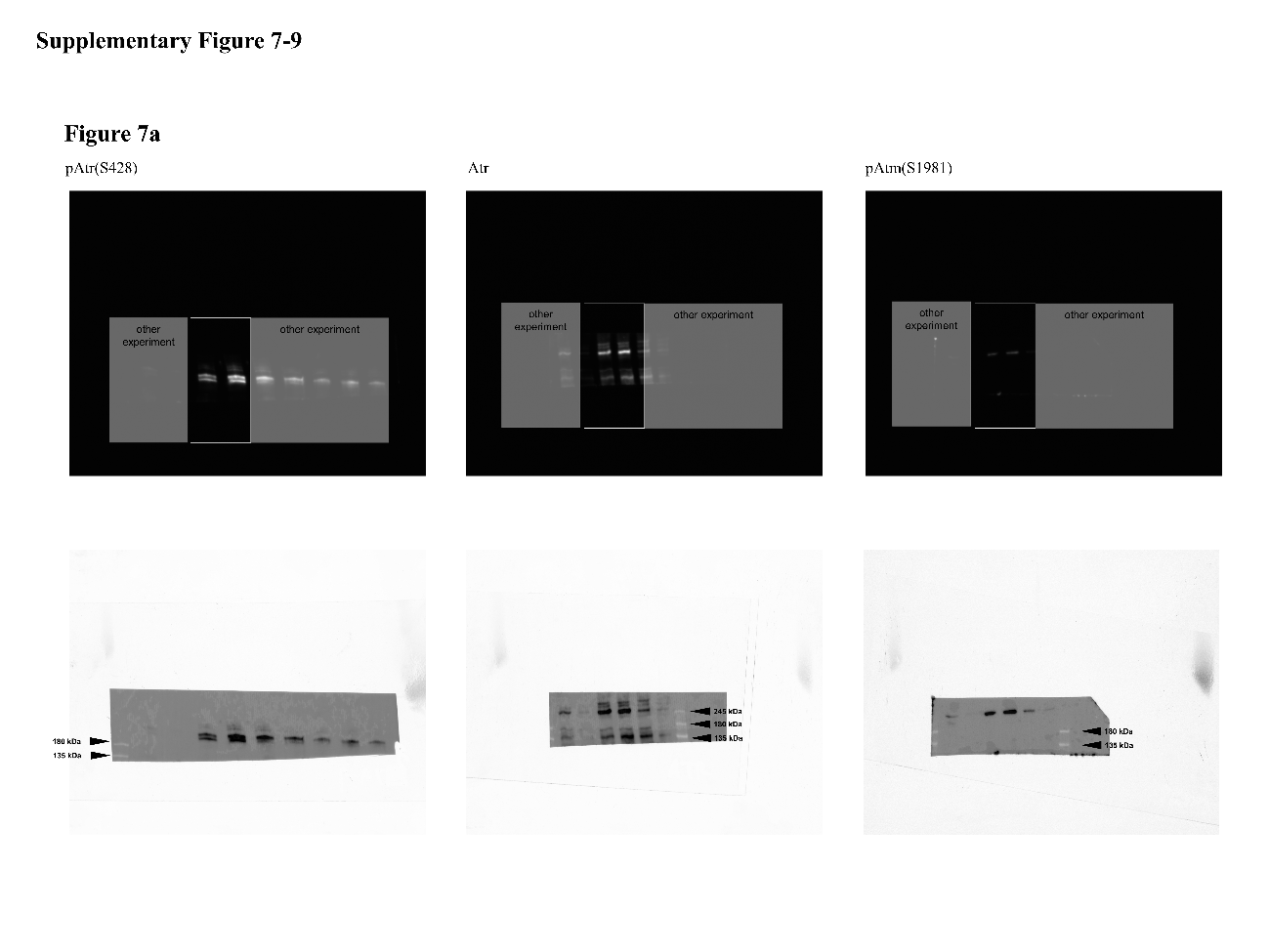


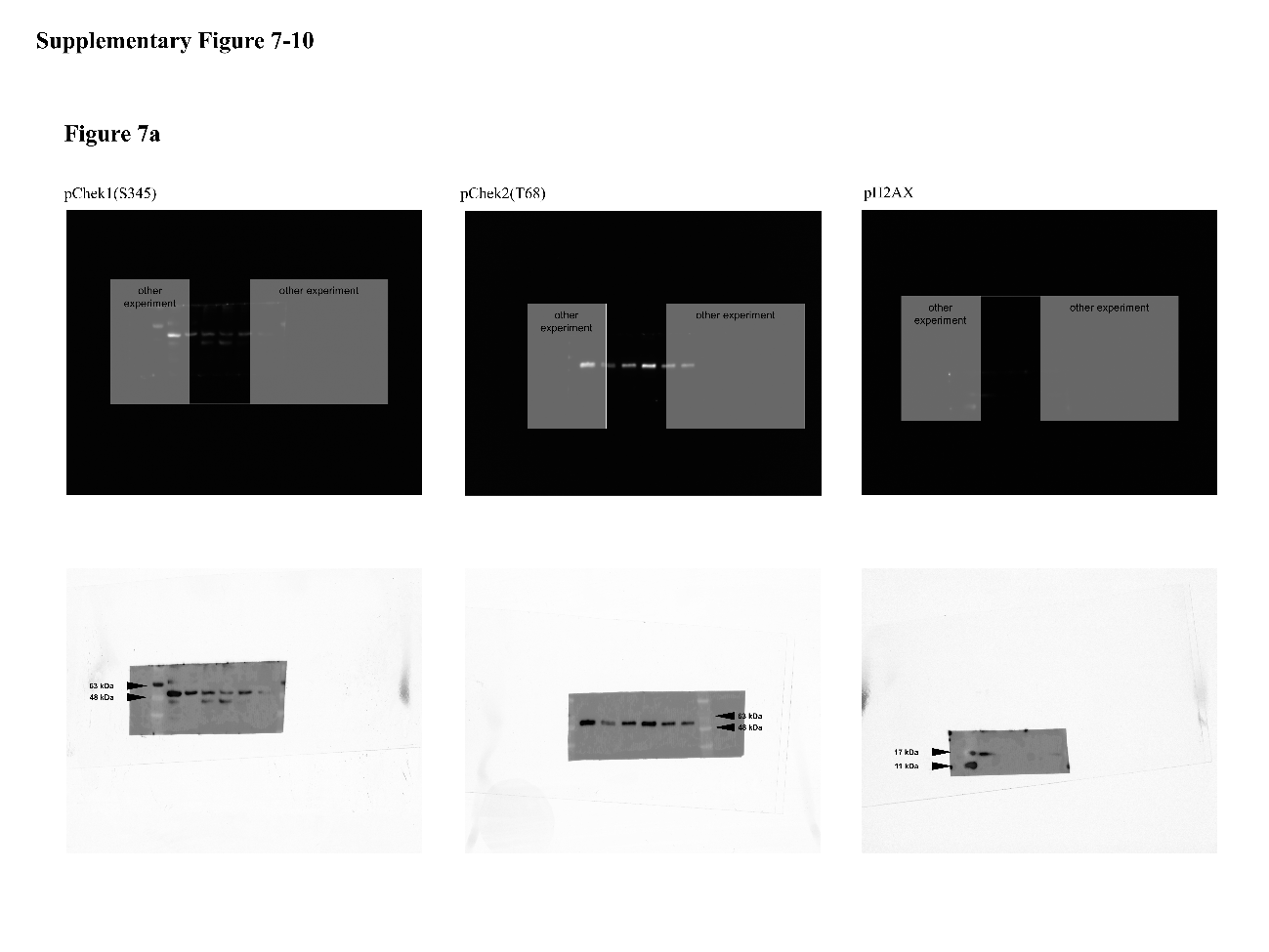


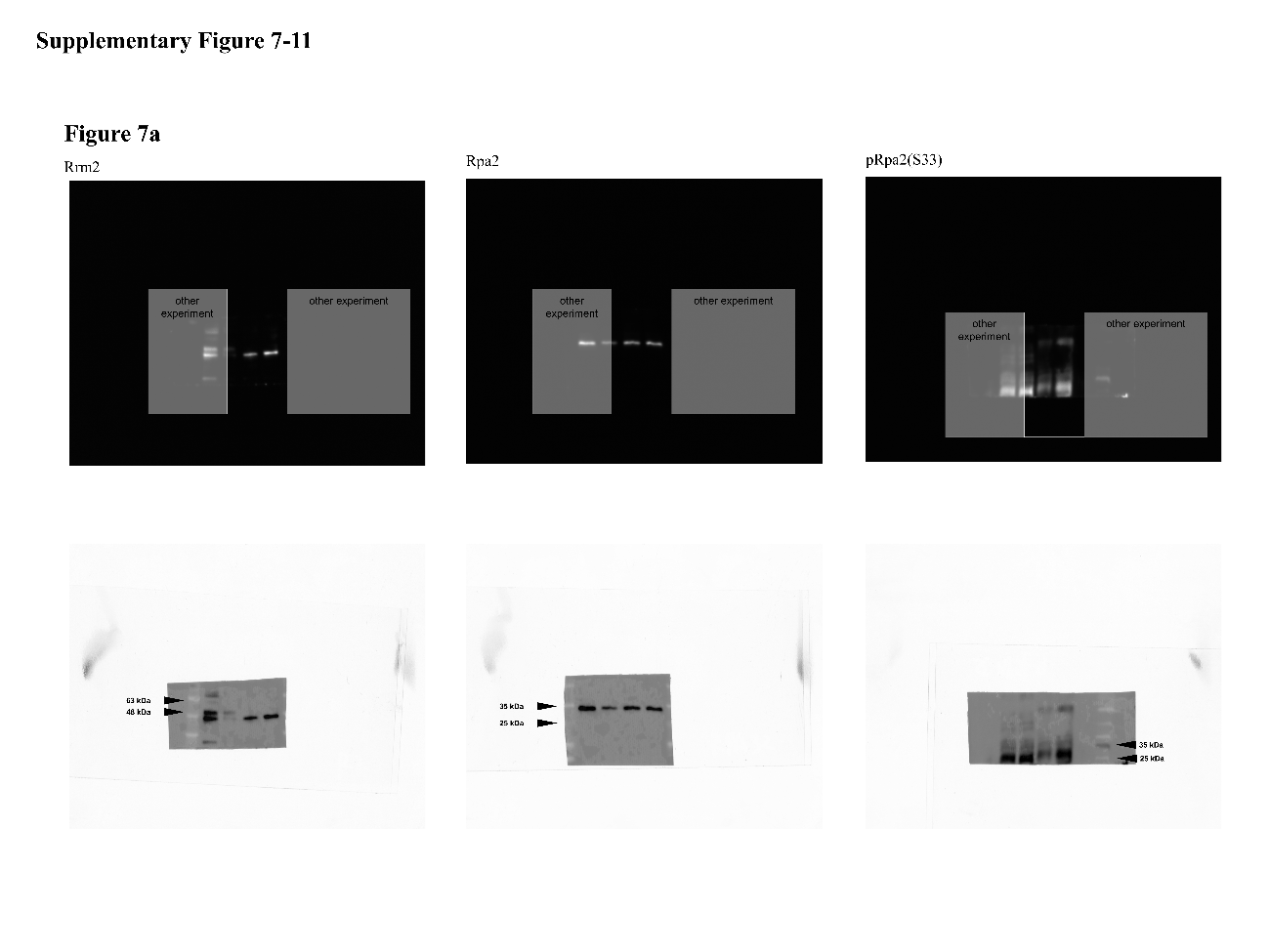


**Supplementary Fig. 7**

All uncropped images are shown. Results other than this study are hidden by shading.
