# Supplementary material for: N(6)-methyladenosine methylation-regulated polo-like kinase 1 cell cycle homeostasis as a potential target of radiotherapy in pancreatic adenocarcinoma
Source: Sci Rep. 2022 Jun 30;12:11074. doi: 10.1038/s41598-022-15196-5 (PMC9246847; doi:10.1038/s41598-022-15196-5)
Supplement: Supplementary file 3 — Supplementary Information 2. [file 41598_2022_15196_MOESM3_ESM.docx]

Supplementary data 2

# FTO_opt amino acid sequence

MKRTPTAEEREREAKKLRLLEELEDTWLPYLTPKDDEFYQQWQLKYPKLILREASSVSEELHKEVQEAFLTLHKHGCLFRDLVRIQGKDLLTPVSRILIGNPGCTYKYLNTRLFTVPWPVKGSNIKHTEAEIAAACETFLKLNDYLQIETIQALEELAAKEKANEDAVPLCMSADFPRVGMGSSYNGQDEVDIKSRAAYNVTLLNFMDPQKMPYLKEEPYFGMGKMAVSWHHDENLVDRSAVAVYSYSCEGPEEESEDDSHLEGRDPDIWHVGFKISWDIETPGLAIPLHQGDCYFMLDDLNATHQHCVLAGSQPRFSSTHRVAECSTGTLDYILQRCQLALQNVCDDVDNDDVSLKSFEPAVLKQGEEIHNEVEFEWLRQFWFQGNRYRKCTDWWCQPMAQLEALWKKMEGVTNAVLHEVKREGLPVEQRNEILTAILASLTARQNLRREWHARCQSRIARTLPADQKPECRPYWEKDDASMPLPFDLTDIVSELRGQLLEAKP*

# FTO_opt DNA sequence

ATGAAGCGCACCCCGACTGCCGAGGAACGGGAAAGAGAGGCCAAGAAGCTGCGGCTGCTGGAAGAACTGGAAGATACCTGGCTGCCCTACCTGACACCTAAGGACGACGAGTTTTACCAGCAGTGGCAGCTGAAGTACCCCAAGCTGATCCTGAGAGAGGCCTCCAGCGTTAGCGAGGAACTGCACAAAGAGGTGCAAGAGGCCTTTCTGACCCTGCACAAGCACGGCTGCCTGTTCAGAGATCTCGTGCGGATCCAGGGCAAAGACCTGCTGACACCCGTGTCCAGAATCCTGATCGGCAATCCCGGCTGCACCTACAAGTACCTGAACACCAGACTGTTCACCGTGCCTTGGCCTGTGAAGGGCAGCAACATCAAGCACACCGAGGCCGAAATTGCCGCCGCTTGCGAGACATTCCTGAAGCTGAACGACTACCTGCAGATCGAGACAATTCAGGCCCTTGAGGAACTGGCCGCCAAAGAGAAAGCCAACGAGGACGCTGTGCCCCTGTGCATGTCTGCCGATTTTCCCAGAGTCGGCATGGGCAGCAGCTACAACGGACAGGATGAGGTGGACATCAAGAGCAGAGCCGCCTACAACGTGACCCTGCTGAACTTCATGGACCCTCAGAAGATGCCTTACCTGAAAGAGGAACCCTACTTCGGAATGGGCAAGATGGCCGTGTCCTGGCACCACGACGAGAACCTGGTGGATAGATCTGCCGTGGCCGTGTACAGCTACTCTTGCGAGGGACCTGAGGAAGAGAGCGAGGACGATAGCCATCTGGAAGGCAGAGATCCCGACATCTGGCACGTGGGCTTCAAGATCAGCTGGGACATCGAGACACCCGGCCTGGCCATTCCACTGCATCAGGGCGATTGCTACTTCATGCTGGACGACCTGAACGCCACACACCAGCATTGTGTGCTGGCCGGCTCTCAGCCTAGATTCAGCAGCACACACAGAGTGGCCGAGTGCAGCACAGGCACCCTGGATTACATCCTGCAGAGGTGTCAGCTGGCCCTGCAGAACGTGTGCGACGACGTGGACAACGACGATGTGTCCCTGAAGTCCTTCGAGCCCGCCGTGCTGAAACAGGGCGAAGAGATCCACAACGAGGTGGAATTCGAGTGGCTGCGGCAGTTCTGGTTCCAAGGCAACCGGTACAGAAAGTGCACCGACTGGTGGTGTCAGCCCATGGCTCAACTGGAAGCCCTGTGGAAGAAAATGGAAGGCGTGACCAATGCCGTGCTGCATGAAGTGAAGCGCGAGGGCCTGCCTGTGGAACAGAGAAACGAGATCCTGACCGCCATCCTGGCCAGCCTGACAGCCAGACAGAATCTGCGGAGAGAATGGCACGCCCGGTGTCAGAGCAGAATCGCCAGAACACTGCCCGCCGACCAGAAACCTGAGTGCAGACCCTACTGGGAGAAAGACGACGCCAGCATGCCCCTGCCTTTCGACCTGACCGATATCGTGTCTGAGCTGAGAGGCCAGCTTCTGGAAGCAAAACCCTAG
